# Kinetic Analysis of Mouse Brain Proteome Alterations Following Chikungunya Virus Infection before and after Appearance of Clinical Symptoms

**DOI:** 10.1371/journal.pone.0091397

**Published:** 2014-03-11

**Authors:** Christophe Fraisier, Penelope Koraka, Maya Belghazi, Mahfoud Bakli, Samuel Granjeaud, Matthieu Pophillat, Stephanie M. Lim, Albert Osterhaus, Byron Martina, Luc Camoin, Lionel Almeras

**Affiliations:** 1 Aix Marseille Université, Unité de Recherche en Maladies Infectieuses et Tropicales Emergentes, UM63, CNRS 7278, IRD 198, Inserm 1095, Marseille, France; 2 Department of Viroscience, Erasmus MC, Rotterdam, The Netherlands; 3 Aix-Marseille Université, CNRS, CRN2M UMR 7286, Marseille, France; 4 CRCM, Marseille Protéomique, Inserm, U1068, Marseille, France; 5 Aix-Marseille Université, UM 105, Marseille, France; 6 Unité de recherche en biologie et épidémiologie parasitaires (URBEP), Institut de Recherche Biomédicale des Armées (IRBA), Marseille, France; University of Liverpool, United Kingdom

## Abstract

Recent outbreaks of Chikungunya virus (CHIKV) infection have been characterized by an increasing number of severe cases with atypical manifestations including neurological complications. In parallel, the risk map of CHIKV outbreaks has expanded because of improved vector competence. These features make CHIKV infection a major public health concern that requires a better understanding of the underlying physiopathological processes for the development of antiviral strategies to protect individuals from severe disease. To decipher the mechanisms of CHIKV infection in the nervous system, a kinetic analysis on the host proteome modifications in the brain of CHIKV-infected mice sampled before and after the onset of clinical symptoms was performed. The combination of 2D-DIGE and iTRAQ proteomic approaches, followed by mass spectrometry protein identification revealed 177 significantly differentially expressed proteins. This kinetic analysis revealed a dramatic down-regulation of proteins before the appearance of the clinical symptoms followed by the increased expression of most of these proteins in the acute symptomatic phase. Bioinformatic analyses of the protein datasets enabled the identification of the major biological processes that were altered during the time course of CHIKV infection, such as integrin signaling and cytoskeleton dynamics, endosome machinery and receptor recycling related to virus transport and synapse function, regulation of gene expression, and the ubiquitin-proteasome pathway. These results reveal the putative mechanisms associated with severe CHIKV infection-mediated neurological disease and highlight the potential markers or targets that can be used to develop diagnostic and/or antiviral tools.

## Introduction

Chikungunya virus (CHIKV), an *Alphavirus* belonging to the *Togaviridae* family, is an arthropod-borne virus transmitted to humans by *Aedes spp*. mosquitoes [1]. Since 2004, major epidemics of Chikungunya fever have occurred in Africa and spread rapidly to India and islands in the southwestern Indian Ocean, notably in La Réunion between 2005 and 2006, when more than one third of the population was affected with the infection, causing 203 deaths [2]. Although *Ae. aegypti* is the classical vector of CHIKV, an adaptive mutation of the virus to *Ae. Albopictus* during the La Réunion outbreak increased the viral transmissibility and dissemination [3]. Climate changes and increased international exchanges of products and people have favored the dissemination of *Ae. albopictus* that colonized the temperate regions of Europe and the Americas [4], [5]. Therefore, the wide distribution of *Ae. albopictus* and its establishment in temperate regions have modified the risk map of CHIKV outbreaks [6]. A recent CHIKV epidemic in northeastern Italy highlighted the increased risk of the emergence of arboviruses transmitted by local competent mosquitoes in Europe [7], [8]. These outbreaks were directly linked to the return of tourists from India and the affected islands in the Indian Ocean. The risk of CHIKV transmission arises from the simultaneous presence of the virus, well-adapted vectors and susceptible human hosts. The spread of the Chikungunya epidemic has caused significant social and economic losses (high economic cost and human suffering) [9]. CHIKV is now considered a global health concern. In the absence of a vaccine or specific treatment, the primary mechanism to protect individuals from CHIKV infection is the prevention of bites from infected *Aedes spp.* using a combination of personal protective measures and vector control strategies [10]. However, protection cannot be restricted to anti-vector measures. Antiviral strategies against CHIKV infection must be developed for the prevention and/or treatment of the clinical manifestations associated with this arboviral disease.

The symptomatology of CHIKV infection was first described in the mid-1950s after an outbreak of Dengue disease in Tanzania in 1952 [11], [12]. Although five percent of the infected people are asymptomatic, the disease is mainly characterized by fever, rash, headache and incapacitating joint pain (arthralgia) [13]. Chikungunya fever is rarely fatal, and most symptoms are resolved within a few weeks; nevertheless, some patients have persistent joint pain in the form of recurrent or persistent episodes that last for months to years [14]. Whereas the neurological complications were described in the 1960s [15], [16], the severe clinical forms involving the central nervous system (CNS) were not uncommon during the Chikungunya outbreak that occurred in La Réunion from March 2005 to April 2006 [17]. This outbreak was characterized by a large number of atypical manifestations, including neurological disorders, which are listed as a major cause of death among individuals with severe CHIKV infection [18]. The increased susceptibility of newborns and the elderly to neurological complications supported the age-dependent association of these severe forms [19]. Additionally, the first mouse model of CHIKV infection developed by Couderc and collaborators [20] revealed the dissemination of the virus to the choroids plexuses and leptomeninges in the CNS in severe infections. This animal model has improved knowledge about the pathogenicity and cell/tissue tropisms of the virus, confirming that CHIKV can disseminate in the CNS.

To prevent and/or treat severe neurological disease in humans, a better understanding of the neurological consequences of CHIKV infection before and after the appearance of neurological clinical symptoms is needed. To elucidate the pathogenesis of CHIKV infection and identify the host factors hijacked by CHIKV to complete its viral replication cycle, the protein profile changes following CHIKV infection *in vitro* and *in vivo* were analyzed using state-of-the art technology. The *in vitro* experiments using CHIKV-infected hepatic or microglial cell lines collected before cell death revealed the down-regulation of host proteins involved in diverse cellular pathways and biological functions, including transcription, translation, cell signaling and lipid and protein metabolism [21], [22]. The *in vivo* experiments comparing the liver and brain protein expression patterns in mock- and CHIKV-infected mouse tissues collected at the peak symptomatic phase showed an alteration of the proteins involved in stress responses, inflammation, metabolism and apoptosis [23]. In contrast to the *in vitro* experiments, most of the differentially expressed proteins in the infected mouse brain tissue were up-regulated. The differences between the global protein expression patterns could be attributed, in part, to the different time points chosen in each proteomics analysis. The *in vitro* experiments focused on early proteome alterations following CHIKV infection, whereas the *in vivo* experiments investigated the molecular consequences of viral infection after the appearance of clinical symptoms.

Therefore, to obtain a comprehensive view of the pathophysiological processes associated with the clinical onset of neurological CHIKV infection, a kinetic analysis was performed on the protein expression profiles in the brain of CHIKV-infected mice collected before and after the onset of clinical symptoms. The host proteome modifications were determined using two proteomic approaches (2D-DIGE and iTRAQ) followed by protein identification by mass spectrometry (MS). Ingenuity Pathway Analysis (IPA) of the total dataset of proteins that were differentially expressed at the early and late time-points enabled the determination of the main networks and pathways modified during CHIKV infection in the brain. Detailed analysis of the proteins involved in these networks and pathways provided insight into the protein interactions and biological processes that are involved in the pathogenesis of the neurological disease caused by CHIKV infection. This study also highlighted the biomarkers of severe atypical symptoms and potential targets for antiviral research.

## Materials and Methods

### Ethics statement

All animal experiments described in this paper have been conducted according to Dutch guidelines for animal experimentation and approved by the Animal Welfare Committee of the Erasmus Medical Centre, Rotterdam, the Netherlands. All efforts were made to minimize animal suffering.

### Reagents

N-hydroxy succinimide ester Cy2, Cy3 and Cy5, urea, glycerol, mineral oil, immobiline DryStrip gels (18 cm, pH = 3–10, pH = 4–7 and pH = 6–11) and IPG buffer solutions (pH = 3–10, 4–7 and 6–11) were purchased from GE Healthcare (Piscataway, NJ). Acrylamide, DTT, Tris, glycine and SDS were purchased from Bio-Rad (Hercules, CA, USA). Dimethyl formamide (DMF), CHAPS, L-lysine, ammonium persulfate, iodoacetamide, agarose, bromophenol blue and TFA were purchased from Aldrich (Poole, Dorset, UK). Thiourea, TEMED, acetone, acetonitrile (ACN) and ethanol were purchased from Fluka (Buchs, Switzerland). Trypsin (sequencing grade) was purchased from Promega (Madison, WI). All buffers were prepared with Milli-Q water (Millipore, Belford, MA, USA). Imperial Protein Stain solution was purchased from Thermo Scientific (Rockford, IL, USA).

### Mouse infection

Nine-day-old female C57/Bl6 mice were infected intra-peritoneally (i.p) with 10^5^ TCID_50_ per mouse of CHIKV strain S27 in 100 μl volumes. Mock infected mice (n = 6) received the same volume of medium and were sacrificed on day 2 post infection. CHIKV-infected mice were sacrificed on the first day that virus was present in the brain (Day 2 post infection; n = 6) and on the first day of neurological symptoms (Day 3 post infection). Two different disease manifestations were observed on day 3 post infection: mice exhibited paralysis-like symptoms (n = 6) or tetanus-like symptoms (n = 6). Brains were collected immediately after humane euthanasia by cervical dislocation under isoflurane anesthesia and cerebellum was separated to reduce protein background. Brains were cut in half and the left hemisphere of each mouse was washed rapidly in ice-cold PBS to remove residual blood contaminants. Brains were then snap-frozen and stored in −80°C until processing. Mice were maintained in isolator cages throughout the infection experiment, had a 12-hour day-night cycle and were fed ad libitum. Animal experiments were approved by the Animal Ethics Committee of Erasmus Medical Center. Virus presence in the brain was confirmed by means of detection of viral RNA and antigen in brains samples from all mice. Viral RNA was extracted from brain samples using the automated MagnaPure method (Total nucleic acid isolation kit, Roche Diagnostics, the Netherlands) according to the manufacturer's instructions, and detected using a one-step RT-PCR TaqMan protocol (EZ-kit, Applied Biosystems) in an ABI PRISM 7500 detection instrument. The primers and probe used for CHIKV RNA detection were: CHIKV-reverse CCAAATTGTCCGGGTCCTCCT; CHIKV-forward AAGCTCCGCGTCCTTTACCAAG and probe Fam-CCAATGTCTTCAGCCTGGACACCTTT-Tamra [24], [25].

Viral antigen was detected in formalin fixed, paraffin embedded tissues as follows: 4-μm thick paraffin sections were deparaffinized in xylene, rehydrated in descending concentrations of ethanol and incubated for 10 min in 3% H_2_O_2_ diluted in PBS to block endogenous peroxidase activity. Antigen retrieval was performed by incubation for 15 min at 121°C in citrate buffer (0.01 M, pH 6.0). Sections were incubated overnight at 4°C with rabbit-anti-CHIKV capsid (1∶5000), antibody followed by secondary goat anti-rabbit IgG-PO antibody (1∶100; Dako, The Netherlands). Sections were counterstained with Mayer's hematoxylin and mounted with Kaiser's glycerin-gelatin and analyzed using a light microscope.

### Protein sample preparation

Half brain hemispheres from non-infected mice and mice infected with CHIKV were collected before and after the appearance of clinical signs corresponding to early and late time-points, respectively. CHIKV-infected mice were separated into three groups: early (n = 6, CH-E1 to E6) sampled at day two and two late groups showing two different symptoms (see above “mouse infection” section for details); late “paralytic” (n = 6, CH-LP1 to LP6) and late “tetanus-like” (n = 6, CH-LT1 to LT6) sampled at day three. Control group (mock, n = 6, C1 to C6) was sampled at day two. Brain samples were stored at −80°C and further processed in biosafety level 3 laboratory (Dept. of Virology, IRBA Marseille) until complete homogenization. Briefly, each brain sample was lysed with 1 ml of lysis buffer containing 2% SDS, 125 mM Tris-HCl pH = 6.8, 10% glycerol and 5% mercaptoethanol, and homogenised by mechanical disruption using metal beads and the Tissue Lyser apparatus (QIAGEN). The resulting homogenates were centrifuged for 15 min at 16 000 x g at 4°C and the supernatant was collected and stored at −80°C. The protein concentration of each sample was determined by the Lowry method (DC Protein assay Kit, Bio-Rad) according to the manufacturer's instructions.

### CyDye labeling

Samples were subjected to 2-D clean-up kit (GE healthcare) and the protein pellet was resuspended in standard cell lysis buffer containing 8 M urea, 2 M thiourea, 4% (w/v) CHAPS and 30 mM Tris, adjusted to pH 8.5 (UTC buffer) at a protein concentration of 2.5 μg/μL. Sample quality and protein amount was checked out by loading 10 μg of each sample onto a 10% SDS-PAGE precast gel (BioRad) stained with Imperial Protein Stain solution (Fisher Scientific) (data not shown). Proteins in each sample were minimally labeled with CyDye according to the manufacturer's recommended protocols and as previously described [26] [27]. An internal standard pool was generated by combining an equal amount of each sample included in the study and was labeled with Cy-2. Cy3-, Cy5- and Cy2-labeled samples were then pooled (Supplementary Tables S1 and S2), and an equal volume of UTC buffer containing 10 mM DTT and 1% (v/v) immobilized pH gradient (IPG) buffer corresponding to the IPG strips used, was added.

### Two-dimensional electrophoresis

Labelled-samples were first separated by isoelectric focusing (IEF) with precast 18-cm IPG strips with different pH gradient ranges (3–10 L, 4–7 or 6–11), followed by a second dimension separation on 10% SDS-PAGE, as previously described [27].

### Image analysis

After electrophoresis, the gels with Cydye-labeled proteins were scanned with a Typhoon Trio Image scanner (GE Healthcare UK). Prescans were performed to adjust the photomultiplier tube (PMT) voltage to obtain images with a maximum intensity of 60 000 to 80 000 U. Images were cropped with ImageQuant software (GE Healthcare UK) and further analyzed using the software package Progenesis SameSpot v2 software (Nonlinear Dynamics, Newcastle upon Tyne, UK). Background subtraction and spot intensity normalization were automatically performed by Progenesis SameSpots. Protein spots which presented a significant abundance variation between the 3 experimental groups (|ratio|≥1.3, ANOVA *p*≤0.05) were marked and submitted to MS for identification.

### In-gel Digestion

Based on the Progenesis SameSpot analysis, protein spots of interest from gels stained with Imperial™ Protein Stain solution were excised and digested using a Shimadzu Xcise automated gel processing platform (Shimadzu Biotech, Kyoto, Japan) as described previously [28] and stored at −20°C until their analysis by MS

### Mass spectrometry analysis of peptide mixture from gel elution and data analysis

The samples were subjected to nanoscale capillary liquid chromatography-tandem mass spectrometry (nano LC-MS/MS) analysis with a QTOF apparatus (Q-TOF Ultima, Waters, MA) as previously described [26]. The peak lists generated in the micromass pkl format, were then fed into a local search engine Mascot Daemon v2.2.2 (Matrix Science, London, UK) against a mixed *Mus musculus* and Chikungunya virus homemade protein database (SwissProt). Search parameters were set in order to allow one missed tryptic cleavage site, the carbamidomethylation of cysteine, and the possible oxidation of methionine; precursor and product ion mass error tolerance was <0.2 Da. All identified proteins have a Mascot score greater than 35 (Mixed: *Mus musculus*, Chikungunya virus, 16487 sequences extracted from Swissprot_2012_02), corresponding to a statistically significant (*p*<0.05) confident identification.

### iTRAQ labeling

For iTRAQ labeling, a sample pool of each experimental group was generated by mixing an equal amount of each sample per group (mock, pool-C; early, pool-E; and late, pool-LP and pool-LT). Six mice per group were pooled. Each pool was then divided into two replicates (mock, pool-C1-C2; early pool-E1-E2 and late pools-LP1-LP2 and LT1-LT2) containing 100 μg of protein. Proteins were precipitated with cold acetone for 2 h at – 20°C, centrifuged for 15 min at 16 000 × *g*, dissolved in 20 μL of Dissolution buffer, denatured, reduced, alkylated and digested with 10 μg of trypsin overnight at 37°C, following manufacturer's protocol (iTRAQ Reagent Multiplex Buffer kit, Applied Biosystems, Foster City, CA, USA) and as previously described [29]. The resulting peptides were labeled with iTRAQ reagents (iTRAQ Reagent-8Plex multiplex kit, Applied Biosystems) according to manufacturer's instructions and as presented in the Supplementary Table S3. Before combining the samples, a pre-mix containing an aliquot of each sample, cleaned-up using a ZipTip was analysed by MS/MS to check out peptide labeling efficiency with iTRAQ reagents and homogeneity of labeling between each sample. The content of each iTRAQ reagent-labeled sample was pooled into one tube according to this previous test. The mixture was then cleaned-up using an exchange chromatography (SCX/ICAT cation exchange cartridge, ABsciex, Foster City, USA) and reverse-phase chromatography C18 cartridge (C18 SpinTips, Proteabio, Nîmes, France), prior to be separated using off-gel system (Agilent 3100 OFFGEL fractionator, Agilent Technologies), as previously described [27].

### Mass spectrometry analysis of peptide fractions from off-gel separation

For nanoLC MS measurements, approximately 5 μg of peptide sample was injected onto a nanoliquid chromatography system (UltiMate 3000 Rapid Separation LC (RSLC) systems, Dionex, Sunnyvale, CA). After pre-concentration and washing of the sample on a Dionex Acclaim PepMap 100 C18 column (2 cm×100 μm i.d. 100 A, 5 μm particle size), peptides were separated on a Dionex Acclaim PepMap RSLC C18 column (15 cm×75 μm i.d., 100 A, 2 mm particle size) (Dionex, Amsterdam) using a linear 90 min gradient (4–40% acetonitrile/H20; 0.1% formic acid) at a flow rate of 300 nL/min. The separation of the peptides was monitored by a UV detector (absorption at 214 nm). The nanoLC was coupled to a nanospray source of a linear ion trap Orbitrap MS (LTQ Orbitrap Velos, Thermo Electron, Bremen, Germany). The LTQ spray voltage was 1.4 kV and the capillary temperature was set at 275°C. All samples were measured in a data dependent acquisition mode. Each run was preceded by a blank MS run in order to monitor system background. The peptide masses were measured in a survey full scan (scan range 300–1700 m/z, with 30 K FWHM resolution at m/z = 400, target AGC value of 10^6^ and maximum injection time of 500 ms). In parallel to the high-resolution full scan in the Orbitrap, the data-dependent CID scans of the 10 most intense precursor ions were fragmented and measured in the linear ion trap (normalized collision energy of 35%, activation time of 10 ms target AGC value of 10^4^, maximum injection time 100 ms, isolation window 2 Da and wideband activation enabled). The fragment ion masses were measured in the linear ion trap to have a maximum sensitivity and the maximum amount of MS/MS data. Dynamic exclusion was implemented with a repeat count of 1 and exclusion duration of 37 sec.

### Data analysis

Raw files generated from MS analysis were combined and processed with Proteome Discoverer 1.1 (Thermo Fisher Scientific). This software was used for extraction of MGF files. Protein identification and quantification were carried out using ProteinPilot version 4.0 (Applied Biosytems). The search was performed against the mixed database containing 55536 sequences (54080 sequences from *Mus musculus* (extracted from Uniprot the 13th December 2011) + 1300 sequences from Chikungunya virus, and 156 classical contaminant proteins). Data were processed as described previously [30].

### SDS-PAGE, Blotting, and Analysis Procedures

Immunoblotting with fluorescence-based methods was used to detect both the total protein expression profile and the specific immunoreactive proteins, as described previously [26]. The same protein samples used for proteomic analysis were minimally labeled with CyDye (*i.e.,* Cy3) as described above (see “CyDye Labeling” section). Labeled samples were separated by 10% or 4–20% SDS-PAGE in a Mini-PROTEAN Cell (Bio-Rad) according to the molecular weight of the targeted proteins. Gels were transferred to a nitrocellulose membrane (0.2 μm; GE Healthcare) using a semidry blotting system at 200 mA for 30 min (TE 77 PWR Semi-Dry Transfer Unit, GE Healthcare) [31]. Blots were saturated with 5% nonfat dried milk in PBS containing 0.05% (v/v) Tween 20 (PBS-T-milk) for 1 h. Western blot (WB) analyses were carried out with rabbit mono- or polyclonal antibodies directed against β-arrestin (1∶5000, ARRB1, no. 4674, Cell Signaling Technology, Danvers, MA), GRIP-associated protein (1∶500, GRASP1, no. sc-135681, Santa Cruz Biotechnology, Inc., Santa Cruz, CA), annexin A2 (1∶100, ANXA2, no. sc-9061, Santa Cruz), integrin αV (1∶100, ITGAV, no. 10179, Santa Cruz), myosin phosphatase target subunit 1 (1∶100, MYPT1, no. sc-25618, Santa Cruz), rabaptin-5 (1∶1000, RABEP1, no. sc-15351, Santa Cruz), N-Ras (1∶500, N-Ras, no. sc-519, Santa Cruz), synaptogyrin-3 (1∶1000, SYNGR3, no. sc-68936, Santa Cruz), or with a goat polyclonal antibody directed against γ-aminobutyric acid receptor subunit alpha-1 (1∶100, GABA_A_Rα1 or GABRA1, no. sc-31045, Santa Cruz), diluted in PBS-T-milk and incubated overnight at 4°C. After three washes in PBS-T, primary rabbit antibodies were revealed with ECL Plex goat anti-rabbit IgG Cy5-conjugated secondary antibody (1∶1000, GE Healthcare), and rabbit anti-goat IgG fluorescein isothiocyanate (FITC) conjugate (1∶400, Southern Biotech, Birmingham, AL) was used for the detection of anti-GABA_A_Rα1 goat antibody diluted in PBS-T-milk. All manipulations were protected from light. The gels electrophoresis and immunoblots were scanned using a Typhoon Trio image scanner as mentioned above (see “Image Analysis” section). Immunoreactive bands were analyzed using TotalLab Quant v12.2 software (Nonlinear Dynamics). To evaluate the expression level of the different proteins, immunoreactive band intensities were normalized to the intensities of a global protein pattern labeled with Cy3 as described previously [26]. Band intensities were also corrected for the adjacent background. Differences in the relative abundance of each protein between two independent groups were determined using Student's *t*-test. All differences were considered significant at *p* <0.05 and statistical analysis was performed using GraphPad Prism v5.01 statistical software (GraphPad Software Inc., La Jolla, CA). Standard molecular weight markers were loaded in each gel (Bio-Rad).

### Ingenuity pathway analysis

A dataset containing deregulated proteins obtained from 2D-DIGE and iTRAQ analysis and their corresponding expression values (fold-change and p-values) were uploaded into the IPA software, Inc (http://www.ingenuity.com), taking into account to protein expression evolution according to clinical onsets. Proteins whose expression was significantly deregulated (|fold-change| ≥ 1.3, *p*-value ≤ 0.05), were selected for the analysis. The IPA program uses a knowledgebase (IPA KB) derived from the scientific literature to relate genes or proteins based on their interactions and functions. Ingenuity Pathway Analysis generates biological networks, canonical pathways and functions relevant to the uploaded dataset. A right-tailed Fisher's exact test is used for calculating *p*-values to determine if the probability that the association between the proteins in the dataset and the functional and canonical pathway can be explained by chance alone. The final scores are expressed as negative log of *p*-values and used for ranking. The scores are derived from a *p*-value (score  =  −log (*p*-value)) and indicate the likelihood that focus proteins (*i.e.,* the identified proteins within a network)) are clustered together. Thus, these proteins and their association with the IPA KB were used to generate networks and to perform functional canonical pathway analyses.

## Results

### Virus Infection Conditions

**Figure 1:**
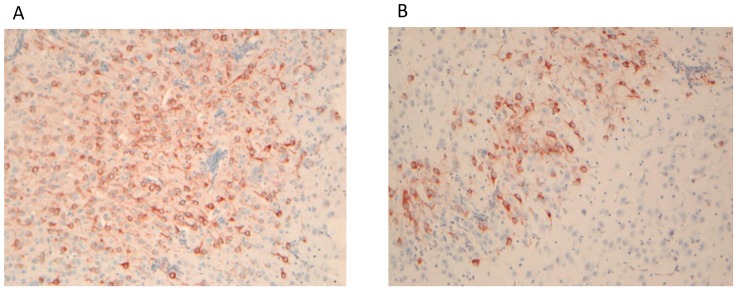
Viral antigen in the brains of CHIKV infected mice. A: CHIKV-antigen present in the cortex of CHIKV-infected mice two days post infection (objective 10x). B: CHIKV-antigen present in the thalamus of infected mice three days post infection (objective 10x).

The presence of the CHIKV RNA in the brains collected from CHIKV-infected mice at the indicated time points was verified by real-time RT-PCR using primers and probes targeting the capsid-encoding region of the viral RNA. The viral RNA was detected on days 2 and 3 post-infection. On day 3, 50- to 100-fold more viral RNA was detected in the brains of infected animals. There was no difference in the RNA level in animals with paralysis and tetanus-like symptoms. Using immunohistochemistry, the viral antigen was detected in the brain of infected animals with paralysis and tetanus-like symptoms (Figure 1). The antigen was detected in the cortex and thalamus of the brain collected at days two and three post-infection. None of the mock-infected mice expressed the viral RNA or antigen.

### 2D-DIGE analysis to detect the differentially expressed proteins following CHIKV infection

**Figure 2:**
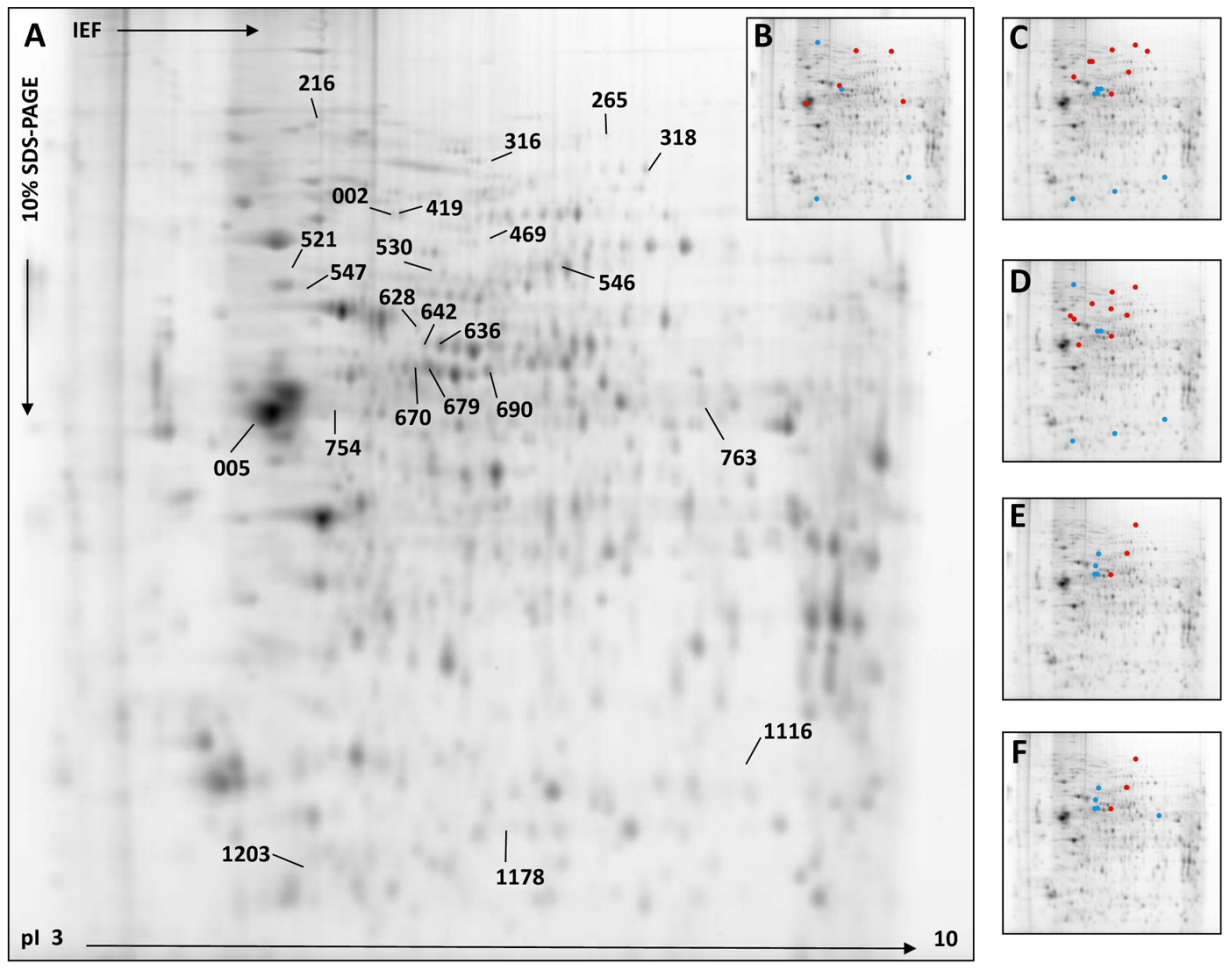
2D-DIGE analysis (pH 3–10) of mock (M)-, early (E), late paralytic (LP) and late tetanus-like (LT) CHIKV-infected brain samples. (A) Representative data from a 2D-DIGE experiment using a 10% SDS-polyacrylamide gel with the pH 3–10 range is shown. Proteins from M-, E- and LP- and LT- CHIKV-infected brain samples were labeled with Cy5 or Cy3 cyanine dyes, as described in Table S1. As determined by Progenesis SameSpot software, protein spots that were differentially regulated between the four experimental groups (|FC| ≥1.3 and *p* ≤0.05), were submitted to mass spectrometry for identification. The numbers annotated on the gel corresponded to master gel numbers of deregulated protein spots. Spots were all identified as *Mus musculus* proteins and were listed in Table 1. Spots differentially modified between E and mock- (B), LP- and E- (C), LT and E- (D), LP and M- (E) and LT and M- (F) infected samples are represented by red (up-regulated) or blue (down-regulated) dots.

Using the Progenesis SameSpot v.2 software, the abundance of 23 protein spots was found to be significantly different (ANOVA, *p*-value ≤ *0.05*) among the four groups (M, E, LP and LT) with fold changes ≥ 30% (|FC| ≥ 1.3, in the pH range 3–10; Figure 2A). The majority of the protein spots were significantly altered at the late time points in the infected mice relative to the mock-infected mice (LP *vs* M, n = 15; eight up-regulated and seven down-regulated, and LT *vs* M, n = 15, nine up-regulated and six down-regulated, Figures 2C and 2D). Nine protein spots were significantly different in the infected mice at the early time point relative to the mock-infected group (five up-regulated and four down-regulated, Figure 2B). Finally, seven and eight protein spots were significantly different in the LP and LT samples, respectively, relative to the early samples (LP *vs* E, three up-regulated and four down-regulated; and LT *vs* E, three up-regulated and five down-regulated, Figures 2E and 2F). Notably, among the modified protein spots, few differences were observed between the LP and LT samples relative to either the mock or early samples.

To better understand the early pathophysiological processes following CHIKV infection and to improve the determination of host proteome changes before the appearance of clinical signs, 2D-DIGE analyses were performed on the mock-infected samples relative to the infected samples at the early time point using narrower pH range IPG strips (*e.g.*, pH 4–7 and 6–11). Using the pH 4–7 IPG strips for the IEF, nine protein spots were found to be significantly different between the early and mock-infected samples (|fold-change| ≥ 1.3, *p* ≤ *0.05*) (seven up-regulated and two down-regulated, Figure S1). However, no protein spots were found to be significantly different using the pH 6–11 IPG strips (data not shown). Considering both pH ranges (*i.e.*, pH 3–10 and 4–7), the abundance of 18 protein spots was found to be significantly modified at the early time point compared to the mock-infected group. Overall, the DIGE analysis of the brain tissue following CHIKV infection revealed that the abundance of 32 protein spots was altered.

### Identification of modified protein abundance following CHIKV infection using 2D-DIGE analyses

Among the 23 protein spots detected using the pH 3–10 range analysis, 18 (78.3%) were successfully identified with a high degree of confidence; these spots corresponded to 16 distinct proteins according to their accession numbers, including seven proteins identified in the early samples (Table 1). These proteins were grouped into functional categories according to their gene ontology (GO). No viral protein was identified. Two spots contained more than one identified protein (#469 and #628), and three proteins could be identified in more than one spot (DPYSL2, DPYSL3 and AL1L1). Five protein spots were not identified, most likely because of insufficient amounts of proteins or low MS spectra qualities.

**Table 1:** Proteins identified from the 2D-DIGE (pH 3–10) analysis of mouse brain lysates collected at early (E), late paralytic (LP) or late tetanus-like (LT) time-points after CHIKV infection.

Accession number(SwissProt)	Protein name	Molecularweight (kDa)	*pI*	Spot ID	Number of MS/MS peptide sequences	Sequence Coverage (%)	Mascot Score	Average volume ratio					
								E *vs* M	LP *vs* E	LP *vs* M	LT *vs* E	LT *vs* M	ANOVA (*p* value)
Host proteins													
Cytoskeleton organization													
CLIP2_MOUSE	CAP-Gly domain-containing linker protein 2	116.35	6.07	316	11	12.0	207	1.3		1.4		1.6	0.003
DYN1_MOUSE	Dynamin-1	98.14	7.61	469	12	15.3	298					1.3	0.011
SPTB2_MOUSE	Spectrin beta chain, brain 1	274.91	5.40	216	18	8.7	298	−1.6				−1.4	0.014
TBB2A_MOUSE	Tubulin beta-2A chain	50.27	4.78	754	2	4.7	728					1.3	1.7e–4
TBB3_MOUSE	Tubulin beta-3 chain	50.84	4.82	005	16	39.8	2226	1.3					0.004
Host response/protein folding/ubiquitination													
GRP78_MOUSE	78 kDa glucose-regulated protein	72.49	5.07	547	7	13.0	183			1.3		1.3	0.006
HS90B_MOUSE	Heat shock protein HSP 90-beta	83.57	4.97	469	7	11.2	362					1.3	0.011
UBE2K_MOUSE	Ubiquitin-conjugating enzyme E2 K	22.51	5.33	1203	3	16.5	131	−1.7		−1.5		−1.7	0.003
VCIP1_MOUSE	Deubiquitinating protein VCIP135	135.67	6.72	265	7	6.8	120		1.3	1.5	1.3	1.5	0.004
Nervous system development													
DPYL1_MOUSE	Dihydropyrimidinase-related protein 1	62.47	6.63	628	4	8.9	166	1.4	−1.7		−1.3		8.8e–4
DPYL2_MOUSE	Dihydropyrimidinase-related protein 2	62.64	5.95	628	9	20.3	326	1.4	−1.7		−1.3		8.8e–4
				636	14	31.1	1137			−1.3		−1.3	0.003
DPYL3_MOUSE	Dihydropyrimidinase-related protein 3	62.30	6.04	670	9	19.3	561		−1.4	−1.3	−1.3		3.1e–4
				679	13	27.9	1035		−1.4	−1.3	−1.3		8.8e–4
				690	14	28.6	1387		1.3	1.4	1.3	1.4	9.0e–6
Metabolic/biosynthetic process													
ACLY_MOUSE	ATP-citrate synthase	120.56	7.13	318	16	16.9	614	1.3		1.3			0.022
AL1L1_MOUSE	Cytosolic 10-formyltetrahydrofolate dehydrogenase	99.50	5.64	002	19	22.9	557			1.3			0.028
				419	25	30.4	741			1.3		1.3	0.019
NDUS1_MOUSE	NADH-ubiquinone oxidoreductase 75 kDa subunit, mitochondrial	80.75	5.51	521	15	21.7	338					1.3	0.012
Transport													
NSF_MOUSE	Vesicle-fusing ATPase	83.13	6.52	546	20	30.9	407		1.3	1.5	1.3	1.5	8.6e–5
Spots not identified													
	n.i.			530					−1.4		−1.3		0.010
	n.i.			642				−1.4		−1.4		−1.3	3.6e–4
	n.i.			763				1.3			1.3		0.003
	n.i.			1116				−1.7		−1.6		−1.8	0.014
	n.i.			1178						−1.4		−1.5	0.010

The proteins were identified by mass spectrometry following in-gel trypsin digestion. The spot numbers correspond to the same numbers as indicated on Figure 2. The identities of the spots, their SwissProt accession numbers, and the theoretical molecular masses and *pI* values as well as the number of peptide sequences, the corresponding percent sequence coverage, and the Mascot score are listed for MS/MS analysis. Protein scores greater than 35 were considered as significant (*p< 0.05*). Paired average volume ratio and *p*-values (ANOVA) between each paired groups compared, were defined using Progenesis Samespot software. n.i., no identification.

M; mock-infected samples.

The nine spots that the pH 4–7 range analysis revealed to be differentially expressed in the infected samples at the early time point were all identified; these spots corresponded to eight distinct host proteins based on their SwissProt accession numbers (Supplementary Table S4). The dynactin subunit 1 (DCTN1) was identified in two spots. Similar to the pH 3–10 analysis, no viral protein was identified. Notably, the early up-regulation of the dihydropyrimidinase-related protein 2 (DPYSL2) was confirmed using the pH 4–7 analysis. Therefore, the use of the narrower pH range enabled the identification of seven proteins in addition to those identified using the pH 3–10 range; therefore, 14 distinct proteins were identified as differentially expressed before the appearance of clinical signs. Considering the results obtained using both pH ranges in the 2D-DIGE analyses and the different paired comparisons, 22 unique host proteins were identified.

### Identification of differentially expressed proteins following CHIKV infection using iTRAQ-labeling

To further characterize the proteome changes in the mouse brain after CHIKV infection, an off-gel quantitative proteomic analysis was performed using the iTRAQ reagent, which allowed the labeling and comparison of 8 different samples (Supplementary Table S3). The data were analyzed with the Protein Pilot software using the parameters described above. More than 3000 proteins were initially identified; following the application of the local False Discovery Rate (FDR) of 5% and the exclusion of contaminants, 2686 proteins identified and quantified, which were included in the analysis.

A total of 178 distinct host proteins were found to be significantly different (*p* ≤ *0.05*) among the four groups (M, E, LP and LT) with a fold-change ≥ 30% (|FC| ≥ 1.3) (Supplementary Table S5). Among these proteins, 129 were differentially expressed in the CHIKV-E- and mock-infected mice, with 87% of the proteins being down-regulated (17 up-regulated and 112 down-regulated). In the CHIKV-LP- and early-infected mice, 138 proteins were differentially expressed with 96% of the proteins being up-regulated (133 up-regulated and five down-regulated). In the CHIKV-LT and early-infected samples, 144 proteins were differentially expressed with more than 98% of the proteins being up-regulated (142 up-regulated and two down-regulated). Among the proteins differentially expressed at the late time-point compared to control samples, 91 proteins were significantly different in the CHIKV-LP- and mock-infected mice (51 up-regulated and 40 down-regulated) and 85 proteins were significantly different in the CHIKV-LT and mock-infected samples (55 up-regulated and 30 down-regulated).

### Combination of in-gel (2D-DIGE) and off-gel (iTRAQ-labeling) analyses

**Figure 3:**
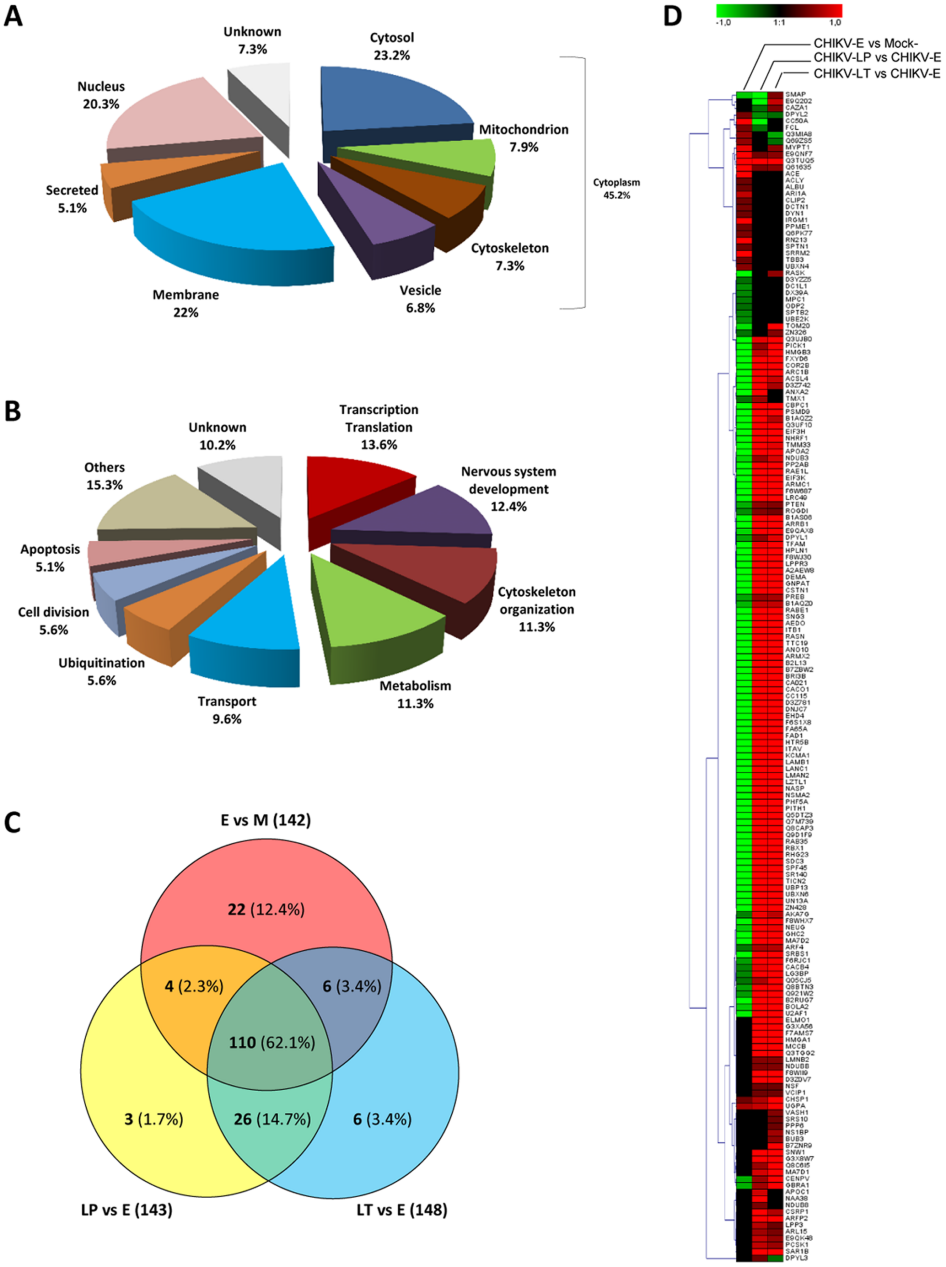
Classification of proteins significantly differentially regulated following CHIKV infection identified by 2D-DIGE and iTRAQ analysis. Significantly differentially regulated proteins were classified according to their sub-cellular location (A) and their functional categorization (B) according to gene ontology. The percentages of proteins associated with each category are indicated in brackets. (C) Venn diagram representing unique host proteins identified according to experimental group comparisons following CHIKV-infection by combined 2D-DIGE and iTRAQ analyses. The number of host proteins significantly differentially regulated between early (E) *vs* mock (M), late paralytic (LP) or late tetanus-like (LT) *vs* E are indicated. The number of proteins associated with each category is indicated with corresponding percentage in brackets. (D) Hierarchical clustering analysis was performed according to the mean ratios calculated between E *vs* M, LP *vs* E and LT *vs* E, as indicated at the top of the graphic. Up- and down-regulated proteins are shown in red and green, respectively, and proteins with no statistically change in expression level are indicated in black. The intensity of red or green color corresponds to the degree of regulation as indicated by the color strip at the top of the figure in arbitrary units. The graphical cluster was generated using the Genesis program [87].

Because the objective of this kinetic study was to determine the protein profile alterations during the evolution of clinical signs, only the proteins that were significantly differentially expressed in the late (LT or LP) and early-infected samples, and in the early-infected and mock samples were included in the combined analysis of the DIGE and iTRAQ data. Considering the times at which the two late symptoms occurred, analysis of the three comparisons (E *vs* M, LP *vs* E and LT *vs* E) generated a total of 177 unique host proteins that were differentially expressed in the brain tissue samples after CHIKV infection at early and/or late time-points (2D-DIGE, n = 17; iTRAQ, n = 161; CRMP1 was detected using both methods). The subcellular distributions and the GO functional classifications were determined for the proteins that were significantly differentially expressed during CHIKV infection. The proteins were located mainly in the cytoplasm (>45%), the membrane (22%) and the nucleus (20%) (Figure 3A). The differentially expressed proteins were mainly involved in transcription/translation (14%), nervous system development (12%), cytoskeleton organization (11%), metabolism (11%) and transport (10%); others were related to cell cycle, ubiquitination or apoptosis (>5%) (Figure 3B). Among the 177 differentially regulated proteins, the majority (n =  110, 62.1%) showed differential expression in all three comparisons, and 36 proteins showed differential expression in paired comparisons (Figure 3C). Finally, a few proteins were found to be differentially expressed in only one comparison (n = 31), particularly in the LP or LT groups compared to the early time-point, corresponding to six and three proteins, respectively. These results highlighted that the number of proteins differentially expressed between the two late time-points (LT and LP) is relatively low. The hierarchical cluster analysis clearly indicated that at the early time-point, the large majority of proteins were down-regulated (80%) and subsequently up-regulated when both the late clinical symptoms occurred (>95%) (Figure 3D).

### Networks, biological pathways and functions involved in the clinical evolution of CHIKV infection in the brain

**Figure 4:**
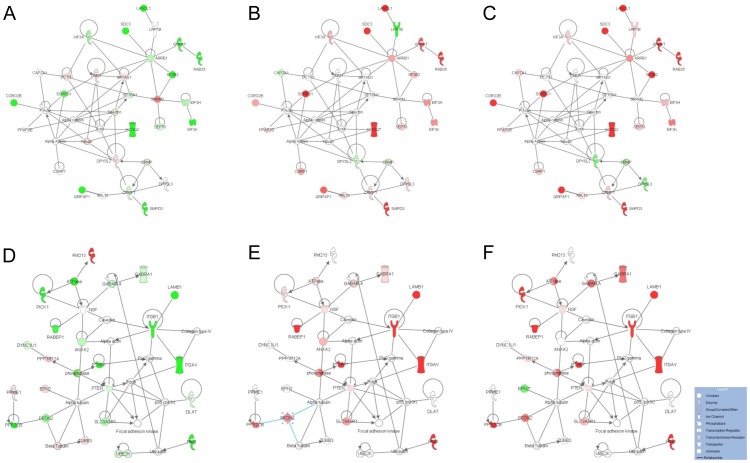
Top-2 most significant protein networks of differentially regulated proteins following CHIKV infection. Ingenuity Pathway Analysis of the total 177 proteins identified as differentially expressed generated 2 emerging networks with high score and including more than 20 molecules with direct relationships. Network 1 (A,B,C) was associated with Cell Morphology, Tissue Morphology, Infectious disease and Network 2 (D,E,F) with Cell-To-Cell Signaling and Interaction, Cellular Assembly and Organization, Cellular Compromise. Each network was overlaid with the protein expression fold-change determined in each separate comparison (early (E) *vs* mock (M) (A, D), late paralytic (LP) *vs* E (B, E) or late tetanus-like (LT) *vs* E (C, F)), to highlight the proteins modified during the time-course of infection. Individual proteins are represented as nodes colored in red and green corresponding to up- and down-regulated proteins, respectively, while the nodes (proteins) in white have been added by IPA to maximize the network connectivity. The different shapes of the nodes represent functional classification of the proteins as indicated in the legend.

The 177 unique host proteins whose levels were found to be significantly altered based on the three comparisons (E *vs* M, LP *vs* E and LT *vs* E) were uploaded into IPA to statistically determine the functions and pathways most strongly associated with the protein list and to establish interactions with other proteins in known networks. Eleven relevant networks were generated by IPA and the top 5 are listed in Table 2. Among these networks, the top two had clearly higher scores (≥ 37) and included more than 20 focus molecules involved in functions related to cell or tissue morphology and infectious disease (network 1); and cell-to-cell signaling and interaction, cellular assembly and organization, and cellular compromise (network 2). These networks were overlaid with the fold changes in protein expression determined in each comparison (E *vs* M, LP *vs* E and LT *vs* E) to highlight the proteins whose levels were altered during the time-course of the infection and according to the late symptoms (Figure 4) [32]. Network 1 contained proteins involved in actin cytoskeleton organization (Actin, Tubulin, Spectrin, CAPZ, and CORO2B), nervous system and recycling machinery (DPYSL, GRIPAP1, and ARRB1) and gene expression regulation (SRRM2, EIF3H, and EIF3K). Network 2 contained proteins related to integrin signaling and cytoskeleton dynamics (ITGAV, ITGB1, and LAMB1), endocytosis and synapse plasticity (GABRA1, PICK1, RABEP1, and NSF), and ubiquitination (RBX1, and RNF213).

**Table 2:** Top-5 networks from IPA of the total 177 proteins differentially expressed identified by 2D-DIGE and iTRAQ labeling following CHIKV infection, considering the 3 comparisons of early (E) *vs* mock (M), late paralytic (LP) or late tetanus-like (LT) *vs* E samples.

Top Functions	Score	Focus Molecules	Molecules in Network
Cell Morphology, Tissue Morphology, Infectious Disease	55	28	Actin, Alpha Actinin, Alpha catenin, **ARL15, ARRB1, CAPZA1, CORO2B, CRMP1**, CRMP, **CSRP1, DCTN1, DNM1, DPYSL2, DPYSL3, EIF3H, EIF3K**, F Actin, **GNPAT, GRIPAP1, KCNQ2, KIF3A, LANCL1, LRP1B, PPAP2B, RAB35, SDC3, SEPT8, SF3B2, SMPD3, SORBS1**, Spectrin, **SPTAN1, SPTBN1, SRRM2, Tubulin**
Cell-To-Cell Signaling and Interaction, Cellular Assembly and Organization, Cellular Compromise	37	22	Alpha actin, Alpha tubulin, **ANXA2**, ATPase, Beta Tubulin, Caveolin, Collagen type IV, **DLAT, DYNC1LI1, EPN2**, Focal adhesion kinase, GABAR-A, **GABRA1, ITGAV, ITGB1, LAMB1, NSF**, p85 (pik3r), Pdgfr, **PFDN2**, phosphatase, **PICK1**, PLC gamma, **PPME1, PPP1R12A, PPP2CB, PTEN, PTPRF, RABEP1, Rbx1, Rnf213, SLC9A3R1, TUBB3, UBE2K**, Ubiquitin
Post-Translational Modification, Cancer, Reproductive System Disease	27	17	ACTC1, **ANO10, ARMCX2, BRI3BP, EML2, FAM65A, FLAD1**, GEMIN4, **HEATR5B**, LPIN1, LPIN2, **LPPR3, LRRC49, MAP7D2, MPC1**, NEK6, NUDT21, **OGFR**, PENK, **PHF5A, PITHD1**, PPAP2A, **PPAP2B**, PPAP2C, PPP2R2D, **PREB**, PRTFDC1, RCC1, RIOK2, SF3B1, SUMO4, TRIM23, TRIP13, **TSTA3**, UBC
Developmental Disorder, Hereditary Disorder, Metabolic Disease	25	16	**ADO, AGTPBP1, APRT, ARMC1**, ATP11A, ATP11B, ATP11C, ATP8A1, CBS, CHMP4A, HPS6, **LMAN2, MB21D2**, MCCC1, **MCCC2**, miR-3974 (miRNAs w/seed AAGGUCA), NYNRIN, PNKP, PPFIA1, PPFIA4, **SLC37A4, SLC9A6**, SPRED1, SUPT5H, **SYNGR3**, TENM2, **TENM3, TMEM30A, TMX1, TTC19**, UBC, **UGP2**, UPF3B, ZFYVE26, **ZNF326**
Cell Cycle, Connective Tissue Disorders, Developmental Disorder	25	16	**ARHGAP23**, ARPC2, ARPC4, ARPC1A, **ARPC1B**, ARPC5L, **ATXN2L, BCL2L13, C11orf58**, C6orf170, **CLSTN1, CNTNAP2**, CORO1B, **HMGB3**, KIFC1, MBTPS1, MCM3AP, **MINK1, NFS1**, NUP107, NUP205, POF1B, RASGRF2, RBM10, **SAR1B**, SLC44A1, SLK, **TMED7**, TMPRSS3, **TPR, U2SURP**, UBC, **UBXN4**, WDR41, **ZFR**

Molecules from the uploaded dataset are indicated in bold. Fold-change expression values of these molecules in each paired comparison are indicated in Tables 1, S4 and S5.

Of the 208 canonical pathways identified, 26 presented a significant association (-Log (*p*-value) >2.0, Supplementary Table S6). The most relevant pathways were related to cell junctions and integrin signaling or associated with endocytosis phenomena (*i.e.*, clathrin-mediated endocytosis (CME) and virus entry via the endocytic pathways) and nervous system signaling (*i.e.*, CDK5, semaphoring signaling in neurons, and neuregulin). In addition, the biological functions associated with the protein dataset, ranked by significance, corresponded to cellular assembly and organization (*p*-value: 1.36E-05; n = 54), cellular function and maintenance (*p*-value: 2.11E-05; n = 49) and the cell cycle (*p*-value: 4.40E-05; n = 32). Furthermore, 53 molecules were associated with cell death and survival (*p*-value: 1.80E-03). In terms of diseases and disorders, 48 proteins were significantly associated with neurological disease (*p*-value: 5.20E-04), consistent with the observation that 27 of the molecules were mostly significantly related to nervous system development and function (*p*-value: 1.68E-04).

### Verification of the differential expression of selected candidate proteins

Among the 177 differentially expressed proteins identified, 9 candidates were selected because they were present in the top 2 networks and the most significant canonical pathways and/or representative of integrin signaling and cytoskeleton dynamics (integrin αV (ITGAV), myosin phosphatase target subunit 1 (MYPT1), NRAS), receptor recycling (arrestin-β1 (ARRB1), GRIP-associated protein (GRIPAP1 or GRASP), annexin A2 (ANXA2), rabaptin-5 (RABEP1)) and synapse function (γ-aminobutyric acid receptor subunit alpha-1 (GABRA1), and synaptogyrin (SYNGR3)). For all WB, each protein sample was labeled with the cyanine-3 dye to reveal any variations in sample loading, which were considered for the normalization and the calculation of the average band volume ratio that was detected by each specific antibody and revealed by a fluorescence-conjugated secondary antibody (FITC or ECL Plex system). Using these conditions, the protein levels during the course of CHIKV infection in the mouse brain were determined.

**Figure 5:**
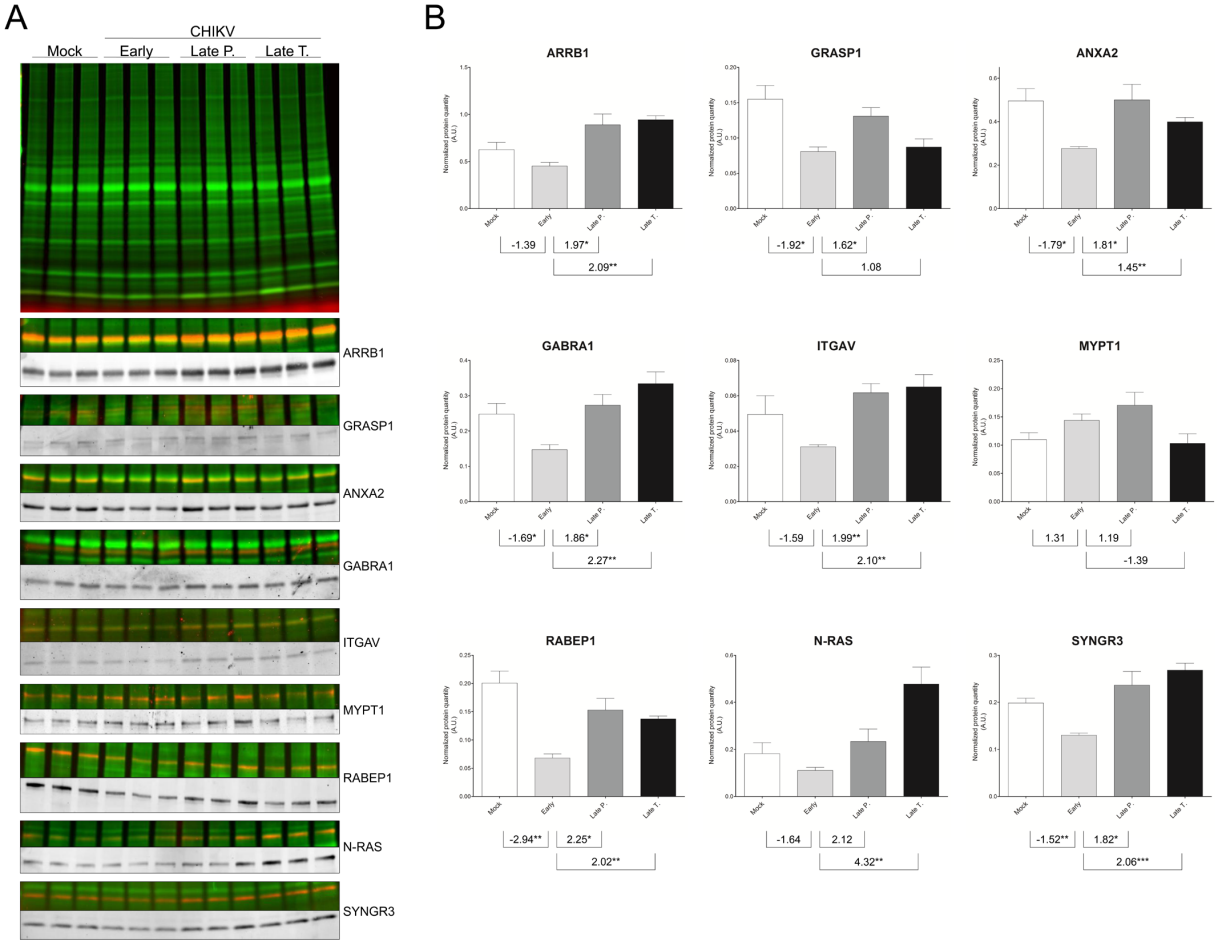
Western blot validations of differentially regulated proteins identified by 2D-DIGE and/or iTRAQ analyses. (A) Protein samples from each group used for proteomic analysis were minimally labeled with cyanine-3 dye. At the top, a representative protein profile of three biological replicates from brain lysates of mock (M), early (E), late paralytic (LP) and late tetanus-like (LT), separated by 10% SDS-PAGE is shown. WB with fluorescence-based methods was used to detect an overlaid fluorescent scan of the general protein patterns (Cy3 dye; green) and the specific immunoreactive proteins (FITC or Cy5 dye; red). To better visualize protein detection signals observed with each specific antibody used, corresponding cropped WB images are presented in grey levels. (B) The graphs correspond to the mean ± S.D. of protein quantity measured by densitometry of the antigenic bands. Densitometry analyses were performed using TotalLab Quant v12.2 software (Nonlinear Dynamics), and data were normalized to levels of global protein pattern intensity. The values indicated under each graph correspond to fold changes from paired comparisons. The significance of the differential protein expression are indicated *, p<0.05; **, p<0.01; ***, p<0.001. A.U., arbitrary units. ANXA2: annexin A2; ARRB1: β-arrestin; GABRA1: γ-aminobutyric acid receptor subunit alpha-1; GRASP1: GRIP-associated protein; ITGAV: integrin αV; MYPT1: myosin phosphatase target subunit 1; N-Ras: N-Ras; RABEP1: rabaptin-5; SYNGR3: synaptogyrin-3.

The significant relative down-regulation between the mock- and early-infected samples and relative up-regulation between the early and LP or LT time points that were detected using the proteomic approaches were confirmed for RABEP1, SYNGR3, GRASP1, ARRB1, GABRA1 and ANXA2 by WB (Figure 5). However, the increased expression of GRASP1 in the LT samples compared to the early-infection samples was not significant in the WB analysis. Although the differential expression of ITGAV and NRAS measured by WB was consistent with the proteomic results according to clinical symptom onset, the protein variations observed were only significantly different between LT or LP *vs* E for ITGAV and between LT *vs* E for NRAS. The MYPT1 differential expression was not significant in the WB analysis, irrespective of the pair-wise comparison performed.

Collectively, for the majority of the selected proteins, the expression variations measured by WB analysis were consistent with the DIGE and iTRAQ analyses over the time course of the clinical symptoms. Nevertheless, the protein abundance changes measured by WB were lower than those detected using the proteomic approaches (Figure 5, Tables 1, S4 and S5). Several previously described factors might affect this validation step [27].

## Discussion

To allow therapeutic intervention of the pathophysiological processes involved in the CHIKV neuro-invasive disease, knowledge of the kinetics of the host protein expression profiles is crucial. Therefore, mouse brain tissues were sampled before and after the appearance of severe clinical symptoms following CHIKV infection, and the protein expression profiles were monitored using comprehensive quantitative proteomic approaches. Unexpectedly, two distinct clinical manifestations were observed in the infected mice, one group showing paralytic symptoms (LP), and the other presenting tetanus-like symptoms (LT). Considering these two clinical signs and focusing the analysis on the alteration of protein profiles, comparisons were limited to successive time points (*i.e.*, E *vs* M, LP *vs* E and LT *vs* E), allowing the determination of 177 unique proteins with significant differential expression.

The most striking results were the opposite trends in differential expression of proteins in the brain during the CHIKV infection in mice. Prior to the appearance of clinical signs, 80% of the proteins that were significantly differentially expressed were down-regulated compared with the mock-infected samples; however, after the appearance of the clinical signs, irrespective of the type of symptom, more than 95% of the proteins that were significantly differentially expressed were up-regulated. The changes in protein expression profiles occurred rapidly, within 24 hr of the onset of the acute phase of the disease. The changes in protein expression profiles were validated by WB analyses for the majority of the selected proteins. The observed contrasting expression profiles are consistent with recent proteomic studies using cell lines or mouse models infected with CHIKV [21], [22], [23]. In CHIKV-infected microglial cells harvested before cellular apoptosis, which was considered to be an early time point, almost all of the proteins that were significantly differentially expressed were down-regulated [21]. In another proteomic analysis, Thio and collaborators reported the down-regulation of 42 out of 50 proteins that were differentially expressed 24 h after the CHIKV infection of hepatic WRL-68 cells; this was considered a model system for early infection time points [22]. Conversely, the analysis of the proteome changes in the brain of mice infected by CHIKV and sampled during the phase of acute neurological symptoms revealed that more than 88% of the proteins were significantly up-regulated [23]. Consistent with these previously reported alterations in the protein expression profiles, the present study clearly indicated that CHIKV infection induces an early dramatic shut-off of host protein expression, followed by an up-regulation during the onset of clinical symptoms. These contrasting protein expression patterns observed using *in vitro* and *in vivo* CHIKV infection models likely reflect the nature of the replication cycle of this virus. Notably, the majority of proteins that were differentially regulated (> 62%) were similar in the three comparisons performed (E *vs* M, LP *vs* E and LT *vs* E), and the level of similarity was as high as 88% at the late time points (*i.e.*, LP *vs* LT). Additionally, in the LP *vs* LT comparison, the expression levels of the common proteins varied in the same direction. These alterations in the protein expression patterns likely reflect the host response in combination with the hijacking of the host protein repertoire for successful viral multiplication and might have important consequences on viral pathogenicity and neurological symptoms.

The *in silico* analysis of proteins that were differentially expressed among the three compared groups (*i.e.*, E *vs* M, LP *vs* E and LT *vs* E) revealed that the main functions and processes altered during the course of CHIKV infection in the mouse brains were as follows: i) integrin signaling and cytoskeleton dynamics, ii) endosome recycling machinery and synapse function, iii) regulation of host gene expression, and iv) modulation of the ubiquitin-proteasome pathway. Several proteins had roles in multiple biological functions, indicating the interconnectedness of these functions. The networks and pathways associated with the differentially expressed proteins are potential pathophysiological processes of neurological CHIKV infection and offer possible biomarkers or therapeutic targets for the diagnosis and prevention of these severe manifestations, as discussed below.

### i) Integrin signaling and cytoskeleton dynamics

**Figure 6:**
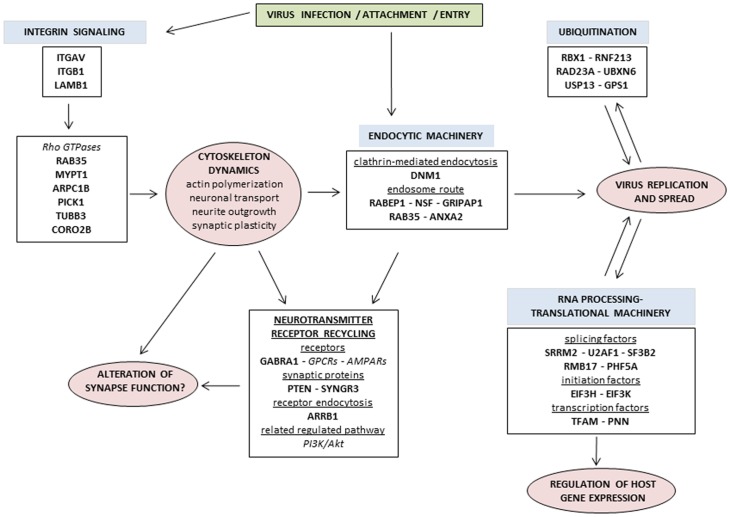
Diagram summarizing the main interconnected pathways and biological processes altered following CHIKV infection in mouse brain. Proteins found to be differentially expressed in the study are shown in bold. Related proteins are shown in italics.

Bioinformatic analysis pointed out that several proteins that were significantly differentially expressed were involved in the integrin signaling cascade and cytoskeleton dynamics, including ITGAV, ITGB1, LAMB1, ARPC1B, CORO2B, RAB35, PICK1, MYPT1, DNM1, and TBB3 (Figure 6). Interestingly, the integrins ITGAV and ITGB1 were found in almost all of the highlighted canonical pathways and in network 2 generated by IPA, suggesting the central role of these membrane proteins. Integrins are composed of alpha and beta subunits and are known to facilitate signal transduction and participate in a variety of processes, including cell growth, blood vessel permeability, tissue repair and immune response [33]. These membrane proteins also function as receptors for diverse viruses and act as signal transducers during virus entry [34]. Although some cell surface proteins are potential receptors for CHIKV entry into the host cells, the precise mechanism is still unclear [35], [36]. In the present study, the down-regulation and up-regulation of αV integrin (ITGAV) and β1 integrin (ITGB1) were observed over the time course of the appearance of clinical signs. The αV/β1 integrin heterodimer is utilized by various adenovirus serotypes for cell entry [37], [38]. In addition, members of the integrin superfamily serve as entry receptors for the Ross River *alphavirus* and the West Nile virus (WNV) [39], [40]. Therefore, it will be interesting to test whether the αV/β1 integrins could be involved in CHIKV attachment or entry into the host cells. In this case, the decrease and subsequent increase in the level of this membrane receptor could first limit and then promote virus entry, respectively. The targeting of integrin receptors is a possible therapeutic strategy against tumor development [41]. Recently, it was shown that the use of αV/β1 antibodies or antagonists prevented the outgrowth or dissemination of carcinoma [42]. Therefore, the candidate drugs targeting αV/β1 integrins could be evaluated for their efficacy in the protection against CHIKV infection or prevention of severe cases.

Notably, the extracellular matrix component LAMB1 was found to be differentially expressed, consistent with previous results using resident brain macrophages infected with CHIKV [21]; LAMB1 can interact with integrin β1, triggering cytoskeleton modifications [43]. The integrin signaling cascade includes the phosphorylation/activation of several protein families, such as the Rho family of small GTPases [44]. Many viruses can hijack the Rho GTPase pathway for efficient virus replication and production [45]. In the present study, the expression levels of several proteins connected to the Rho GTPase pathway were found to be altered (Figure 6), for instance, the Rab GTPase RAB35, a protein that controls the endocytic recycling pathway [46] and participates in the maintenance of cellular adhesion by the inhibition of factors involved in integrin recycling [47]. Disturbance of the equilibrium between these factors could lead to the rupture of cell adhesion, leading to blood vessel permeability and facilitating the crossing of the blood brain barrier by CHIKV.

The altered expression of other proteins involved in microtubule filament formation (ARPC1B) [48], brain cytoskeleton rearrangement/motility (CORO2B, MYPT1) [49], [50] and synaptic plasticity (PICK1) [51] highlighted the importance of changes in cellular cytoskeletal maintenance and molecular trafficking (Figure 6). Alteration of the coronin protein level (CORO1A) in CHIKV-infected brains was previously observed [23].

Other cytoskeleton-related proteins such as dynamin (DNM1) and Tubulin beta-3 chain (TUBB3) were up-regulated only at the early time point. The role of the cytoskeletal network in CHIKV endocytosis has been demonstrated using actin- or tubulin-specific depolymerizing agents, highlighting the importance of intact actin filaments and microtubules for CHIKV infection [52].

### ii) Endosome recycling machinery and synapse function

Similar to the cytoskeletal dynamics-related changes, several proteins that were differentially expressed during the time course of CHIKV infection in the brain were related to the endocytic machinery, including Rabaptin-5 (RABEP1), RAB35, N-ethylmaleimide–sensitive fusion protein (NSF), GRIP-associated protein 1 (GRIPAP1 or GRASP1), and annexin-A2 (ANXA2); this class of proteins was mainly down-regulated at the early stage of infection and then up-regulated at the later time-points (Figure 6). The identification of the differential expression of the proteins involved in different steps of endosome trafficking, such as RABEP1, involved in early endosome fusion [53], [54], NSF, in membrane fusion and transport [53], [55], GRIPAP1, in endosome recycling [56], RAB35, in endosome recycling to the membrane [57], [58] and ANXA2, in different steps from early endocytosis to endosome recycling [59], suggested the strong perturbation of this pathway following CHIKV infection. Diverse viruses including CHIKV use different stages of this endocytic pathway for efficient infection [52], [60], [61], [62], [63], [64], [65]. Endosome trafficking appears to play a key role for the CHIKV replication cycle, and the proteins identified in the present study could represent potential drug targets for the control of viral spreading. Drugs licensed for human use targeting the Rab GTPase network are already available [66]; these drugs could be tested against CHIKV infections.

Several differentially expressed proteins identified in the CHIKV-infected brain samples were associated with neurotransmitter receptor recycling, which is involved in synapse functions (Figure 6). The γ-aminobutyric acid receptor subunit alpha-1 (GABAAR1 or GABRA1), which mediates post-synaptic transmission in the vertebrate CNS, presented an early down-regulation and late up-regulation with the onset of clinical signs. The differential expression of GABRA1 is associated with the modulation of the phosphatase and tensin homolog (PTEN), a synaptic signaling protein [67] and arrestin-b1 (ARRB1), both of which activate the PI3K pathway, leading to the regulation of GABRA1 membrane expression and function [68], [69]. ARRB1, NSF, PICK1 and GRIPAP1 were related to the endocytosis/recycling process of other neurotransmitter receptors (GPCRs or AMPARs) [56], [70], [71], [72]. In addition, the synaptic vesicle protein synaptogyrin (SYNGR3), which is involved in neurotransmission [73], [74], was also found to be differentially expressed in this study. The alteration of these protein levels in CHIKV-infected brains indicates a profound impairment of receptor signaling, leading to synapse dysfunction. The resulting alteration of neurotransmission may be, in part, associated with severe neurological CHIKV infection, as observed in patients with HIV encephalitis with differential expression of the genes encoding the neuronal molecules involved in synaptic plasticity, including synaptogyrin [75]. Further studies are needed to precisely determine the balance between receptor endocytosis, late recycling to the membrane and degradation and the effect on synaptic plasticity from early to severe CHIKV infection. GABAAR are targets of several anti-seizure pharmacological compounds such as benzodiazepines [76]. The potential beneficial effect of these molecules in neurological cases of CHIKV infection should be investigated.

Together, these data showed that the endosomal machinery was affected by CHIKV infection in the brain, which could facilitate virus entry and spread and potentially cause the dysregulation of synapse function and neurotransmission (Figure 6).

### iii) Regulation of gene expression

Because viral genomes are small with limited protein encoding ability, viruses require host transcriptional and translational machineries to complete their replication cycle. Furthermore, the *alphaviruses*, including CHIKV, induce the cessation of host transcription/translation in infected cells [77], [78], [79]. Here, several proteins involved in mRNA processing and translation, including transcription factors (TFAM, PNN), splicing factors (SRRM2, SF32B2, U2AF1, RMB17, PHF5A,) and translation initiation factors (EIF3H and EIF3K) were differentially expressed (Figure 6). Apart from SRRM2 and PNN, all these proteins were down-regulated and up-regulated at early and late time points (*i.e.*, LT and LP), respectively. The early down-regulation of proteins regulating gene expression and/or translation was reported in previous proteomic studies using CHIKV-infected cells [21], [22]. The differential expression of splicing and translation proteins, including EIF3, has also been detected following infection with other viruses [26], [80].

Altogether, these data supported that CHIKV infection, similar to infection with other viruses, perturbed the host protein synthesis machinery at different levels: transcription/translation and maturation, which could explain the down-regulation and up-regulation of the differentially expressed proteins at the early and late stages of the infection, respectively (Figure 6). Further studies on these regulators of gene expression will elucidate their contribution to CHIKV pathogenesis.

### iv) Modulation of the ubiquitin-proteasome pathway (UPP)

The ubiquitin-proteasome pathway (UPP) is indispensable for a variety of cellular processes including the control of protein stability, protein trafficking, the regulation of signal transduction pathways and antiviral responses [81], [82]. Conversely, the hijacking of the UPP by diverse viruses prevents viral protein degradation, aiding evasion of the host immune response, and also enables viral replication, viral particle assembly and egress [83]. In this study, six UPP-associated proteins were differentially expressed during the neurological CHIKV infection (*i.e.*, RBX1, RNF213, RAD23A, GPS1, USP13 and UBXN6) (Figure 6). Ring box protein-1 (RBX1) and Ring finger protein 213 (RNF213) are E3 ubiquitin-ligases that function in protein ubiquitination. The opposing directions of differential expression of the two E3 ubiquitin-ligases before and after the onset of clinical symptoms indicated the nuanced regulation of these proteins. Because E3 ligases are the components of the ubiquitin cascade that confer substrate specificity, knowledge of their specific host and/or viral protein targets might clarify whether the observed E3 ligase expression patterns are more beneficial for the pathogen or the host. The alteration of E3 ligase expression was previously observed in the brain of mice infected with WNV [27], indicating that the expression of these proteins is particularly perturbed after viral infection. The nucleotide excision repair protein RAD23A interacts with the 19S subunit of the 26S proteasome to deliver poly-ubiquitinated proteins to the proteasome for degradation. Li and collaborators showed that in HIV-infected macrophage cells, the HIV-1 viral protein R (Vpr) interacts with the proteasome through RAD23A to promote host protein degradation [84]. The decreased expression of the RAD23A protein in macrophages led to a decrease in HIV replication, indicating the crucial role of RAD23A in viral multiplication. Li et al. suggested that the interaction between Vpr, RAD23A and the 26S proteasome could lead to the degradation of antiviral factors [84]. Finally, two de-ubiquitinating enzymes (DUBs), ubiquitin carboxyl-terminal hydrolase 13 (USP13) and ubiquitin regulatory X domain-containing protein-6 (UBXN6), were down-regulated and up-regulated, respectively, before and after the onset of clinical symptoms in mice infected with CHIKV. This is the first report of the differential expression of DUBs following an *alphavirus* infection. The consequences of the differential expression of the DUBs need to be explored to clarify their involvement in CHIKV infection. The large number of proteins from the UPP that are differentially expressed following CHIKV infection highlights the crucial role of this pathway. Recently, the rapid down-regulation of the UPP proteins was observed following CHIKV infection in a hepatic cell line; however, none of the same proteins were detected relative to this study [22]. Notably, several molecules targeting E3-ligases, DUBs or the proteasome are candidates for cancer treatment [85], which highlights novel potential therapeutic strategies targeting the UPP in CHIKV infection.

## Conclusion

The analysis of differentially protein expression during the time course of CHIKV infection indicated the profound down-regulation of proteins in the early stage, followed by a rapid up-regulation after the appearance of clinical symptoms, consistent with the previously reported dramatic cessation of protein expression in CHIKV-infected cells. These analyses revealed that the differentially expressed proteins were involved in several biological processes including i) cell signaling via integrins, which is related to cytoskeletal dynamics, ii) endocytosis and receptor recycling, which are associated with virus circulation and synapse function, iii) the host translational machinery, which reflects the regulation of protein expression, and iv) modulation of the ubiquitin-proteasome pathway. By revealing the protein expression profiles correlated with the onset of clinical symptoms, this study paves the way for further studies on the evaluation of potential disease markers and the development of new therapeutic targets to prevent the CHIKV neurological infection.

## Supporting Information

Figure S1 **2D-DIGE analysis (pH 4–7) of mock-(M) and early (E) CHIKV-infected brain samples.** Representative data from a 2D-DIGE experiment using a 10% SDS-polyacrylamide gel with the pH 4–7 range is shown. Proteins from mock- and early- CHIKV-infected brain samples were labeled with Cy3 and Cy5 cyanine dyes, respectively. As determined by Progenesis SameSpot software, protein spots that were differentially regulated between the two experimental conditions (|FC| ≥1.3 and *p*≤0.05) were submitted to mass spectrometry for identification. The numbers annotated on the gel corresponded to master gel numbers of deregulated protein spots. All spots were identified as *Mus musculus* and are were listed in the supplementary Table S4. Red and blue numbers correspond to up- and down- regulated spots, respectively.
(TIF)

Table S1 **Experimental design for the 2D-DIGE analysis using pH 3–10 IEF.**
(DOC)

Table S2 **Experimental design for the 2D-DIGE analysis using pH 4–7 or 6–11 IEF.**
(DOC)

Table S3 **Experimental design for iTRAQ reagent-labelling of brain sample pools.**
(DOC)

Table S4 **Proteins identified from the differential 2-D DIGE (pH 4–7) analysis of mouse brain lysates collected at early- compared to mock-group after CHIK- infection.**
(DOC)

Table S5 **Dataset of proteins identified by iTRAQ labeling and tandem mass spectrometry as differentially expressed between mock-(M), early-(E) and late paralytic (LP) or late tetanus-like (LT) CHIKV-infected samples, indicating fold-changes and** ***p*** **-values in each comparison, and GO subcellular location and biological function.**
(DOC)

Table S6 **Ingenuity Canonical Pathways showing a strong significant association [-Log(p-value) >2.0] using the total dataset of 177 proteins differentially expressed in the 3 observations (early (E) vs mock (M), late paralytic (LP) vs E and late tetanus-like (LT) vs E).**
(DOC)
