# Supplementary figures and images for: Kinetic Analysis of Mouse Brain Proteome Alterations Following Chikungunya Virus Infection before and after Appearance of Clinical Symptoms
Source: PLoS One. 2014 Mar 11;9(3):e91397. doi: 10.1371/journal.pone.0091397 (PMC3949995; doi:10.1371/journal.pone.0091397)

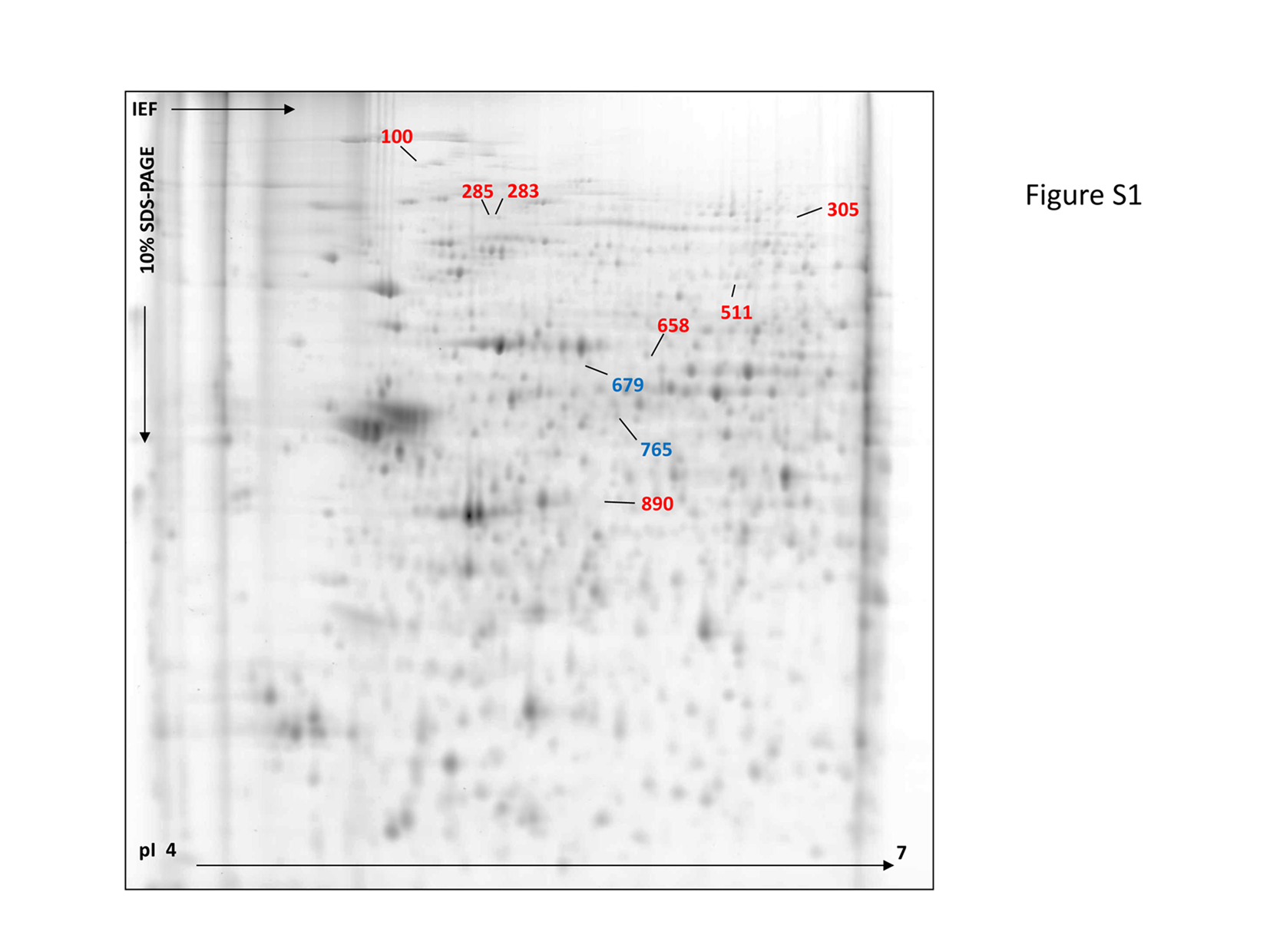

Supplement: Figure S1 — 2D-DIGE analysis (pH 4–7) of mock-(M) and early (E) CHIKV-infected brain samples. Representative data from a 2D-DIGE experiment using a 10% SDS-polyacrylamide gel with the pH 4–7 range is shown. Proteins from mock- and early- CHIKV-infected brain samples were labeled with Cy3 and Cy5 cyanine dyes, respectively. As determined by Progenesis SameSpot software, protein spots that were differentially regulated between the two experimental conditions (|FC| ≥1.3 and p≤0.05) were submitted to mass spectrometry for identification. The numbers annotated on the gel corresponded to master gel numbers of deregulated protein spots. All spots were identified as Mus musculus and are were listed in the supplementary Table S4. Red and blue numbers correspond to up- and down- regulated spots, respectively. (TIF) [file pone.0091397.s001.tif]
