# Supplementary material for: Kinetic Analysis of Mouse Brain Proteome Alterations Following Chikungunya Virus Infection before and after Appearance of Clinical Symptoms
Source: PLoS One. 2014 Mar 11;9(3):e91397. doi: 10.1371/journal.pone.0091397 (PMC3949995; doi:10.1371/journal.pone.0091397)
Supplement: Table S1 — Experimental design for the 2D-DIGE analysis using pH 3–10 IEF. (DOC) [file pone.0091397.s002.doc]

**Table S1.** Experimental design for the 2D-DIGE analysis using pH 3-10 IEF. Mock- (control), early (E) and Late (LP or LT) CHIKV-infected mice brain samples were labeled with cyanine 3 (Cy3) or cyanine 5 (Cy5). An internal standard pool was generated by combining equal amounts of each sample tested in this study, and labeled with Cy2.

| **Gel number** | **Uninfected mice**  **(Ctrl=C)** | **Mice infected by *CHIK* and**  **brain collected at day 2**  **(early=E)** | **Mice infected by *CHIK* and**  **brain collected atday 3**  **(late “para”=LP)*** | **Mice infected by *CHIK* and**  **brain collected at day 3**  **(late “teta”=LT)**** |
| --- | --- | --- | --- | --- |
| 1 | Cy5-C1 | Cy3-CHIK-E1 |  |  |
| 2 | Cy3-C2 |  | Cy5-CHIK-LP1 |  |
| 3 | Cy5-C3 |  |  | Cy3-CHIK-LT3 |
| 4 |  | Cy5-CHIK-E2 | Cy3-CHIK-LP2 |  |
| 5 |  | Cy3-CHIK-E3 |  | Cy5-CHIK-LT4 |
| 6 |  |  | Cy5-CHIK-LP3 | Cy3-CHIK-LT5 |
| 7 | Cy3-C4 | Cy5-CHIK-E4 |  |  |
| 8 | Cy5-C5 |  | Cy3-CHIK-LP4 |  |
| 9 | Cy3-C6 |  |  | Cy5-CHIK-LT6 |
| 10 |  | Cy3-CHIK-E5 | Cy5-CHIK-LP5 |  |
| 11 |  | Cy5-CHIK-E6 |  | Cy3-CHIK-LT1 |
| 12 |  |  | Cy3-CHIK-LP6 | Cy5-CHIK-LT2 |

* CHIKV-infected mice presented paralytic symptoms at day 3 (late “para”=LP)

** CHIKV-infected mice presented tetanus-like symptoms at day 3 (late “teta”=LT)
