# Supplementary material for: Kinetic Analysis of Mouse Brain Proteome Alterations Following Chikungunya Virus Infection before and after Appearance of Clinical Symptoms
Source: PLoS One. 2014 Mar 11;9(3):e91397. doi: 10.1371/journal.pone.0091397 (PMC3949995; doi:10.1371/journal.pone.0091397)
Supplement: Table S2 — Experimental design for the 2D-DIGE analysis using pH 4–7 or 6–11 IEF. (DOC) [file pone.0091397.s003.doc]

**Table S2.** Experimental design for the 2D-DIGE analysis using pH 4-7 or 6-11 IEF. Mock- (control), early (E) CHIKV-infected mice brain samples were labeled with cyanine 3 (Cy3) or cyanine 5 (Cy5). An internal standard pool was generated by combining equal amounts of each sample tested in this study, and labeled with Cy2.

| **Gel number** | **Uninfected mice**  **(ctrl=C)** | **Mice infected by *CHIK* and**  **brain collected at day 2**  **(early=E)** |
| --- | --- | --- |
| 1 | Cy5-C1 | Cy3-CHIK-E1 |
| 2 | Cy3-C2 | Cy5-CHIK-E2 |
| 3 | Cy5-C3 | Cy3-CHIK-E3 |
| 4 | Cy3-C4 | Cy5-CHIK-E4 |
| 5 | Cy5-C5 | Cy3-CHIK-E5 |
| 6 | Cy3-C6 | Cy5-CHIK-E6 |
