# Supplementary material for: Kinetic Analysis of Mouse Brain Proteome Alterations Following Chikungunya Virus Infection before and after Appearance of Clinical Symptoms
Source: PLoS One. 2014 Mar 11;9(3):e91397. doi: 10.1371/journal.pone.0091397 (PMC3949995; doi:10.1371/journal.pone.0091397)
Supplement: Table S3 — Experimental design for iTRAQ reagent-labelling of brain sample pools. (DOC) [file pone.0091397.s004.doc]

**Table S3.** Experimental design for iTRAQ reagent-labelling of brain sample pools. A pool was generated for each group by mixing equal protein amount of each individual sample. Each group pool was divided into two replicates containing 100 µg protein - Mock-CHIKV infected (C1, C2), WNV-infected and collected at early time point (CH-E1, CH-E2) or at late time point considering the two clinical symptoms (CH-LP1, CH-LP2, CH-LT1, CH-LT2*) - digested with trypsin and the resulting peptides of each sample were specifically labeled with one iTRAQ reagent as indicated below.

| **Sample pools** | | **Isobaric iTRAQ**  **reagent** | | --- |   **)** |
| --- | --- | --- |
| C1 | 113 |
| C2 | 114 |
| CH-E1 | 115 |
| CH-E2 | 116 |
| CH-LP1 | 117 |
| CH-LP2 | 118 |
| CH-LT1 | 119 |
| CH-LT2 | 121 |

* CHIKV-infected mice presented at day 3 either paralytic symptoms (LP) or tetanus-like symptoms (LT)
