# Supplementary material for: Kinetic Analysis of Mouse Brain Proteome Alterations Following Chikungunya Virus Infection before and after Appearance of Clinical Symptoms
Source: PLoS One. 2014 Mar 11;9(3):e91397. doi: 10.1371/journal.pone.0091397 (PMC3949995; doi:10.1371/journal.pone.0091397)
Supplement: Table S4 — Proteins identified from the differential 2-D DIGE (pH 4–7) analysis of mouse brain lysates collected at early- compared to mock-group after CHIK- infection. (DOC) [file pone.0091397.s005.doc]

**Table S4**. Proteins identified from the differential 2-D DIGE (pH 4-7) analysis of mouse brain lysates collected at early- compared to mock-group after CHIK- infection.

| **Accession number**  **(Swissprot)** | **Protein name** | **Molecular**  **weight (kDa)** | ***pI*** | **Spot**  **ID** | **Number of MS/MS**  **peptide sequences** | **Sequence**  **Coverage (%)** | **Mascot**  **score** | **CHIKV E vs M** |  |
| --- | --- | --- | --- | --- | --- | --- | --- | --- | --- |
|  |  |  |  |  |  |  |  | **Average volume ratio** | **(*p* value)** |
| **Host proteins** |  |  |  |  |  |  |  |  |  |
| **Cytoskeleton orgnaization** |  |  |  |  |  |  |  |  |  |
| DCTN1_MOUSE | Dynactin subunit 1 [Mus musculus] | 142.27 | 5.66 | 285 | 16 | 14.4 | 320 | 1.3 | 0.016 |
|  |  |  |  | 283 | 11 | 10.6 | 318 | 1.4 | 0.005 |
| DYN1_MOUSE | Dynamin-1 [Mus musculus] | 98.14 | 7.61 | 511 | 15 | 18.7 | 362 | 1.3 | 0.02 |
| SPTA2_MOUSE | Spectrin alpha chain, brain [Mus musculus] | 285.22 | 5.20 | 100 | 2 | 0.9 | 115 | 1.3 | 0.038 |
| **Metabolic/biosynthetic process** |  |  |  |  |  |  |  |  |  |
| ODP2_MOUSE | Dihydrolipoyllysine-residue acetyltransferase component of pyruvate dehydrogenase complex, mitochondrial [Mus musculus] | 68.47 | 8.81 | 679 | 7 | 13.2 | 153 | -1.3 | 0.015 |
| PPME1_MOUSE | Protein phosphatase methylesterase 1 [Mus musculus] | 42.63 | 5.67 | 890 | 1 | 3.1 | 66 | 1.3 | 3.84e-04 |
| **Host response/ptotein folding** |  |  |  |  |  |  |  |  |  |
| ALBU_MOUSE | Serum albumin [Mus musculus] | 70.70 | 5.75 | 305 | 3 | 8.7 | 142 | 1.3 | 0.015 |
| **Cell division** |  |  |  |  |  |  |  |  |  |
| DC1L1_MOUSE | Cytoplasmic dynein 1 light intermediate chain 1 [Mus musculus] | 56.86 | 6.01 | 765 | 4 | 9.0 | 86 | -1.7 | 0.031 |
| **Nervous system development** |  |  |  |  |  |  |  |  |  |
| DPYL2_MOUSE | Dihydropyrimidinase-related protein 2 [Mus musculus] | 62.64 | 5.95 | 658 | 9 | 19.4 | 321 | 1.6 | 0.024 |

The proteins were identified by mass spectrometry following in-gel trypsin digestion. The spot numbers correspond to the same numbers as indicated on Figure S1. The identities of the spots, their SwissProt accession numbers, and the theoretical molecular masses and *pI* values as well as the number of peptide sequences, the corresponding percent sequence coverage, and the Mascot score are listed for MS/MS analysis. Protein scores greater than 35 were considered as significant (*p< 0.05*). Paired average volume ratio and *p* values (Student *t-test*) between each pair groups compared, were defined using Progenesis Samespot software. *n.i*., no identification.
