# Supplementary material for: Kinetic Analysis of Mouse Brain Proteome Alterations Following Chikungunya Virus Infection before and after Appearance of Clinical Symptoms
Source: PLoS One. 2014 Mar 11;9(3):e91397. doi: 10.1371/journal.pone.0091397 (PMC3949995; doi:10.1371/journal.pone.0091397)
Supplement: Table S5 — Dataset of proteins identified by iTRAQ labeling and tandem mass spectrometry as differentially expressed between mock-(M), early-(E) and late paralytic (LP) or late tetanus-like (LT) CHIKV-infected samples, indicating fold-changes and p -values in each comparison, and GO subcellular location and biological function. (DOC) [file pone.0091397.s006.doc]

**Table S5.** Dataset of proteins identified by iTRAQ labeling and tandem mass spectrometry as differentially expressed between mock-(M), early-(E) and late paralytic (LP) or late tetanus-like (LT) CHIKV-infected samples, indicating fold-changes and p-values in each comparison, and GO subcellular location and biological function.

| **Protein name** | **Uniprot KB ID** | **Gene** | **E vs M** | | **LP vs E** | | **LT vs E** | | **LP vs M** | | **LT vs M** | | **Localisdation** | **Function/Process** |
| --- | --- | --- | --- | --- | --- | --- | --- | --- | --- | --- | --- | --- | --- | --- |
|  |  |  | **Fold-change** | **p-value** | **Fold-change** | **p-value** | **Fold-change** | **p-value** | **Fold-change** | **p-value** | **Fold-change** | **p-value** |  |  |
| Pinin | Q3TUQ5_MOUSE | Pnn | 10.00 | 1.63E-02 | 6.89 | 2.52E-02 | 4.94 | 2.64E-02 | 10.00 | 1.42E-02 | 10.00 | 1.46E-02 | Membrane | Transcription |
| Contactin-associated protein-like 2 | E9QNF7_MOUSE | Cntnap2 | 5.65 | 1.93E-02 | 1.34 | 3.56E-03 | 1.64 | 2.40E-03 |  |  |  |  | Membrane | Nervous System |
| GTP-binding protein (Interferon gamma inducible protein 47, isoform CRA_a) | Q61635_MOUSE | Ifi47 | 3.36 | 5.69E-03 | 1.44 | 3.89E-02 | 1.59 | 1.52E-02 | 4.40 | 4.07E-03 | 3.14 | 1.98E-02 | Cytoplasm | Response |
| UTP--glucose-1-phosphate uridylyltransferase | UGPA_MOUSE | Ugp2 | 2.02 | 1.77E-02 | 2.33 | 1.41E-02 | 3.45 | 2.03E-03 | 14.19 | 4.41E-04 | 10.00 | 5.11E-04 | Cytoplasm | Metabolism |
| Calcium-regulated heat stable protein 1 (CRHSP-24) | CHSP1_MOUSE | Carhsp1 | 1.31 | 1.21E-02 | 2.10 | 1.83E-03 | 4.73 | 1.54E-03 | 3.53 | 9.08E-03 | 10.00 | 2.60E-03 | Cytoplasm | Transcription |
| Atp2b1 protein (Fragment) | Q05CJ5_MOUSE | Atp2b1 | *-1.38* | 8.49E-03 | 2.09 | 1.88E-02 | 3.18 | 1.30E-02 |  |  |  |  | Membrane | Transport |
| ADP-ribosylation factor 4 | ARF4_MOUSE | Arf4 | *-1.43* | 1.69E-02 | 2.06 | 7.89E-03 | 2.14 | 1.77E-03 |  |  |  |  | Vesicle | Nervous system |
| A-kinase anchor protein 7 isoform gamma | AKA7G_MOUSE | Akap7 | *-1.53* | 1.55E-02 | 2.84 | 3.66E-03 | 2.22 | 3.79E-03 |  |  |  |  | Membrane | Metabolism |
| Voltage-dependent L-type calcium channel subunit beta-4 | CACB4_MOUSE | Cacnb4 | *-1.54* | 2.16E-02 | 2.77 | 3.37E-02 | 4.71 | 2.44E-02 | 1.75 | 1.20E-02 | 3.84 | 3.26E-03 | Membrane | Nervous system |
| Galectin-3-binding protein | LG3BP_MOUSE | Lgals3bp | *-1.60* | 2.79E-02 | 3.78 | 3.65E-03 | 6.55 | 1.63E-03 | 3.12 | 3.53E-03 | 4.15 | 2.45E-03 | Membrane | Receptor |
| Protein rogdi homolog | ROGDI_MOUSE | Rogdi | *-1.68* | 1.70E-03 | 1.56 | 4.41E-04 | 1.36 | 1.35E-02 |  |  |  |  | Nucleus | Cell division |
| Tial1 cytotoxic granule-associated RNA binding protein-like 1. isoform CRA_a | Q921W2_MOUSE | Tial1 | *-1.86* | 1.52E-03 | 6.18 | 1.03E-02 | 7.15 | 1.00E-02 | 1.48 | 9.13E-03 | 1.53 | 1.04E-02 | Nucleus | Cell division |
| Teneurin-3 (Fragment) | F6RJC1_MOUSE | Odz3 | *-1.90* | 2.24E-02 | 4.50 | 2.04E-02 | 6.75 | 1.52E-02 | *-1.67* | 8.45E-03 |  |  | Membrane | Unknown |
| DNA segment. Chr 16. human D22S680E. expressed. isoform CRA_c (Protein D16H22S680E) | Q8BTN3_MOUSE | D16H22S680E | *-1.91* | 8.11E-03 | 6.62 | 1.69E-03 | 8.39 | 9.04E-03 | 2.65 | 2.93E-03 | 2.49 | 8.08E-03 | Mitochondrion | Unknown |
| Prolactin regulatory element-binding protein | PREB_MOUSE | Preb | *-1.99* | 1.11E-02 | 1.85 | 8.48E-03 | 1.98 | 9.65E-04 | *-1.30* | 9.27E-03 |  |  | Nucleus | Transcription |
| Phosphatidylinositol-3.4.5-trisphosphate 3-phosphatase and dual-specificity protein phosphatase PTEN | PTEN_MOUSE | Pten | *-2.09* | 3.53E-02 | 1.73 | 5.22E-04 | 1.53 | 1.38E-02 | 1.34 | 1.35E-02 |  |  | Cytoplasm | Apoptosis |
| Centromere protein V | CENPV_MOUSE | Cenpv | *-2.10* | 2.61E-02 | 1.85 | 1.53E-02 | 6.61 | 1.35E-02 |  |  |  |  | Nucleus | Cell division |
| Gamma-aminobutyric acid receptor subunit alpha-1 (GABA(A) receptor subunit alpha-1) | GBRA1_MOUSE | Gabra1 | *-2.11* | 1.77E-02 | 1.92 | 5.88E-04 | 6.87 | 5.31E-04 |  |  | 3.52 | 4.23E-03 | Membrane | Nervous system |
| Septin 8 | B1AQZ0_MOUSE | sept8 | *-2.18* | 9.80E-03 | 2.36 | 6.26E-03 | 2.31 | 1.05E-02 |  |  | *-1.50* | 1.01E-02 | Secreted | Cell cycle |
| Small acidic protein | SMAP_MOUSE | Smap | *-2.43* | 4.50E-02 | -3.21 | 2.49E-02 | 1.48 | 2.56E-02 | *-5.59* | 2.95E-02 |  |  | Unknown | Unknown |
| Neurogranin | NEUG_MOUSE | Nrgn | *-2.43* | 1.55E-02 | 3.69 | 8.62E-03 | 2.84 | 8.25E-04 | 1.49 | 6.67E-03 |  |  | Cytoplasm | Nervous system |
| BolA-like protein 2 | BOLA2_MOUSE | Bola2 | *-2.43* | 3.39E-02 | 10.00 | 1.83E-02 | 10.00 | 1.66E-02 |  |  | *-1.39* | 4.31E-02 | Unknown | Unknown |
| Sodium/hydrogen exchanger (Fragment) | E9QAX8_MOUSE | Slc9a6 | *-2.55* | 3.36E-02 | 3.50 | 1.76E-02 | 4.52 | 1.91E-02 |  |  |  |  | Membrane | Transport |
| NADH dehydrogenase [ubiquinone] 1 beta subcomplex subunit 3 | NDUB3_MOUSE | Ndufb3 | *-2.57* | 1.87E-03 | 1.84 | 2.14E-02 | 2.58 | 4.54E-04 |  |  |  |  | Mitochondrion | Metabolism |
| Splicing factor U2AF 35 kDa subunit | U2AF1_MOUSE | U2af1 | *-2.89* | 3.68E-02 | 9.54 | 1.76E-02 | 9.55 | 1.44E-02 | *-1.62* | 4.65E-02 | *-1.57* | 4.69E-02 | Nucleus | RNA processing |
| Protein quaking (MqkI) (qkI) | F8WHX7_MOUSE | Qk | *-2.95* | 3.27E-02 | 4.63 | 1.70E-02 | 3.48 | 1.97E-02 |  |  |  |  | Nucleus | Translation |
| Transcription factor A. mitochondrial | TFAM_MOUSE | Tfam | *-3.11* | 3.42E-02 | 2.81 | 2.18E-02 | 4.19 | 1.37E-02 | *-10.00* | 2.46E-02 | *-2.00* | 4.40E-02 | Mitochondrion | Transcription |
| Beta-arrestin-1 | ARRB1_MOUSE | Arrb1 | *-3.57* | 1.24E-03 | 4.51 | 2.27E-02 | 5.64 | 1.79E-02 |  |  |  |  | Cytoplasm | Apoptosis |
| Na(+)/H(+) exchange regulatory cofactor NHE-RF1 | NHRF1_MOUSE | Slc9a3r1 | *-3.65* | 3.86E-02 | 4.32 | 2.49E-02 | 2.99 | 2.52E-02 |  |  |  |  | Membrane | Cytoskeleton organization |
| Eukaryotic translation initiation factor 3 subunit H | EIF3H_MOUSE | Eif3h | *-3.75* | 4.31E-03 | 5.44 | 1.22E-03 | 3.52 | 1.03E-02 | *-3.41* | 1.35E-02 | *-4.51* | 1.09E-02 | Cytoplasm | Translation |
| Zinc finger RNA binding protein | B2RUG7_MOUSE | Zfr | *-4.03* | 3.46E-02 | 9.55 | 1.38E-02 | 10.00 | 1.92E-02 |  |  |  |  | Nucleus | Developmental protein |
| Transmembrane protein 33 (Protein DB83) | TMM33_MOUSE | Tmem33 | *-4.61* | 4.41E-02 | 5.73 | 2.42E-02 | 2.71 | 2.53E-02 |  |  |  |  | Membrane | Unknown |
| 2-aminoethanethiol dioxygenase | AEDO_MOUSE | Ado | *-4.72* | 8.68E-03 | 5.06 | 3.53E-03 | 4.47 | 2.23E-03 | *-1.45* | 1.63E-02 |  |  | Cytoplasm | Metabolism |
| Non-histone chromosomal protein HMG-17 (Fragment) | F6W687_MOUSE | Hmgn2 | *-4.75* | 1.77E-03 | 3.36 | 8.57E-03 | 3.32 | 1.59E-03 | *-1.90* | 1.06E-02 | *-1.66* | 1.33E-02 | Nucleus | Transcription |
| Kinesin family member 3A (Kinesin-like protein KIF3A) | B1AQZ2_MOUSE | Kif3a | *-4.76* | 1.22E-02 | 3.90 | 2.72E-03 | 2.17 | 3.87E-03 | *-2.17* | 9.11E-03 | *-2.21* | 1.77E-02 | Cytoplasm | Cytoskeleton organization |
| Lipid phosphate phosphatase-related protein type 3 | LPPR3_MOUSE | Lppr3 | *-4.83* | 6.68E-03 | 4.77 | 1.07E-03 | 5.85 | 9.58E-03 | 1.80 | 3.57E-03 |  |  | Membrane | Metabolism |
| Isoform 3 of Cytosolic carboxypeptidase 1 | CBPC1_MOUSE | Agtpbp1 | *-5.09* | 4.34E-02 | 4.00 | 2.72E-02 | 2.97 | 2.57E-02 | 3.25 | 4.07E-02 | 2.28 | 3.69E-02 | Nucleus | Nervous System |
| Crmp1 protein | Q6P1J1_MOUSE | Crmp1 | *-5.31* | 1.17E-02 | 5.15 | 1.17E-02 | 6.65 | 3.93E-04 | *-1.38* | 7.80E-03 |  |  | Cytoplasm | Nervous system |
| Prefoldin subunit 2 (Fragment) | F8WJ30_MOUSE | Pfdn2 | *-5.46* | 3.26E-02 | 5.21 | 1.77E-02 | 6.60 | 1.93E-02 |  |  |  |  | Cytoplasm | Protein folding |
| Mitochondrial glutamate carrier 2 | GHC2_MOUSE | Slc25a18 | *-5.67* | 1.23E-03 | 10.00 | 4.75E-03 | 10.00 | 3.71E-04 |  |  |  |  | Mitochondrion | Transport |
| Armadillo repeat-containing protein 1 | ARMC1_MOUSE | Armc1 | *-6.27* | 3.21E-03 | 4.19 | 2.05E-03 | 4.28 | 2.07E-03 | *-4.18* | 7.25E-03 |  |  | Cytoplasm | Transport |
| Isoform 2 of Leucine-rich repeat-containing protein 49 | LRC49_MOUSE | Lrrc49 | *-6.73* | 4.68E-03 | 5.27 | 2.66E-03 | 5.52 | 1.87E-03 | 2.66 | 9.01E-03 | 2.67 | 1.64E-02 | Cytoskeleton | Cytoskeleton organization |
| Sorbin and SH3 domain-containing protein 1 (Ponsin) | SRBS1_MOUSE | Sorbs1 | *-6.81* | 3.13E-03 | 10.00 | 8.04E-04 | 10.00 | 1.37E-03 |  |  |  |  | Membrane | Metabolism |
| Hyaluronan and proteoglycan link protein 1 | HPLN1_MOUSE | Hapln1 | *-7.12* | 3.66E-03 | 6.55 | 9.22E-03 | 8.12 | 9.54E-03 | -1.71 | 1.83E-02 | -1.98 | 1.76E-02 | Secreted | Cell adhesion |
| Discs. large (Drosophila) homolog-associated protein 3 | B1AS06_MOUSE | Dlgap3 | *-7.17* | 2.64E-03 | 8.08 | 5.43E-03 | 10.00 | 7.19E-04 | -1.42 | 1.45E-02 |  |  | Cytoplasm | Nervous system |
| High mobility group protein B3 | HMGB3_MOUSE | Hmgb3 | *-7.33* | 2.95E-02 | 2.29 | 2.24E-02 | 5.79 | 1.74E-02 | -8.66 | 3.27E-02 | -10.00 | 3.46E-02 | Nucleus | Development |
| MAP7 domain-containing protein 2 | MA7D2_MOUSE | Map7d2 | *-7.62* | 5.54E-03 | 10.00 | 1.50E-02 | 10.00 | 1.60E-03 |  |  |  |  | Unknown | Unknown |
| Opioid growth factor receptor | OGFR_MOUSE | Ogfr | *-8.11* | 3.94E-02 | 6.52 | 2.41E-02 | 4.00 | 2.66E-02 |  |  |  |  | Membrane | Receptor |
| 26S proteasome non-ATPase regulatory subunit 9 (26S proteasome regulatory subunit p27) | PSMD9_MOUSE | Psmd9 | *-8.47* | 4.66E-03 | 5.87 | 9.06E-03 | 4.62 | 1.96E-02 | *-1.62* | 4.69E-02 |  |  | Cytoplasm | Transcription |
| Rab GTPase-binding effector protein 1 (Rabaptin-5) | RABE1_MOUSE | Rabep1 | *-8.65* | 2.76E-02 | 10.00 | 1.37E-02 | 10.00 | 1.36E-02 |  |  |  |  | Vesicle | Endocytosis |
| Synaptogyrin-3 | SNG3_MOUSE | Syngr3 | *-8.82* | 7.52E-04 | 10.00 | 2.02E-04 | 10.00 | 2.33E-04 |  |  |  |  | vesicle | Nervous system |
| Dihydroxyacetone phosphate acyltransferase | GNPAT_MOUSE | Gnpat | *-9.48* | 5.23E-03 | 8.10 | 1.53E-03 | 10.00 | 1.22E-03 | *-1.85* | 3.86E-02 |  |  | Vesicle | Nervous system |
| Apolipoprotein A-II. isoform CRA_a | APOA2_MOUSE | Apoa2 | *-9.49* | 3.76E-02 | 5.96 | 2.25E-02 | 8.74 | 2.89E-02 | *-10.00* | 3.61E-02 | *-1.58* | 4.33E-02 | Secreted | Transport |
| Integrin beta-1 | ITB1_MOUSE | Itgb1 | *-9.86* | 2.84E-02 | 10.00 | 1.34E-02 | 9.59 | 1.37E-02 | 2.61 | 2.45E-02 | *-9.77* | 2.84E-02 | Membrane | Apoptosis |
| Splicing factor 3b. subunit 2 | Q3UJB0_MOUSE | Sf3b2 | *-10.00* | 9.19E-03 | 2.75 | 1.51E-02 | 10.00 | 4.60E-04 | *-6.31* | 6.27E-03 | 2.38 | 3.62E-03 | Nucleus | RNA processing |
| EH domain-containing protein 4 | EHD4_MOUSE | Ehd4 | *-10.00* | 1.26E-02 | 10.00 | 1.11E-02 | 10.00 | 9.17E-03 | *-3.02* | 1.79E-02 |  |  | Membrane | Endocytosis |
| mRNA export factor | RAE1L_MOUSE | Rae1 | *-10.00* | 4.42E-02 | 6.75 | 3.53E-02 | 9.92 | 2.54E-02 |  |  |  |  | Nucleus | Response |
| Calsyntenin-1 | CSTN1_MOUSE | Clstn1 | *-10.00* | 3.08E-03 | 8.77 | 1.64E-02 | 10.00 | 1.14E-03 |  |  |  |  | Membrane | Nervous system |
| GTPase NRas (Transforming protein N-Ras) | RASN_MOUSE | Nras | *-10.00* | 3.29E-02 | 10.00 | 3.33E-02 | 9.60 | 2.88E-02 |  |  |  |  | Membrane | Cytoskeleton organization |
| PHD finger-like domain-containing protein 5A | PHF5A_MOUSE | Phf5a | *-10.00* | 2.59E-02 | 10.00 | 1.32E-02 | 10.00 | 1.33E-02 |  |  | *-7.87* | 2.59E-02 | Nucleus | Transcription |
| Protein unc-13 homolog A | UN13A_MOUSE | Unc13a | *-10.00* | 1.18E-02 | 10.00 | 2.87E-04 | 10.00 | 2.00E-02 | 1.78 | 1.64E-01 | *-1.98* | 5.74E-03 | Membrane | Nervous system |
| FXYD domain-containing ion transport regulator 6 (PLM-like protein) (Phosphohippolin) | FXYD6_MOUSE | Fxyd6 | *-10.00* | 4.39E-02 | 4.59 | 2.89E-02 | 10.00 | 2.44E-02 | *-1.46* | 3.89E-02 | 3.94 | 2.79E-02 | Membrane | Transport |
| Dematin (Erythrocyte membrane protein band 4.9) | DEMA_MOUSE | Epb49 | *-10.00* | 1.71E-02 | 8.25 | 1.04E-02 | 10.00 | 8.49E-04 | *-2.60* | 1.10E-02 | 3.11 | 2.13E-03 | Cytoplasm | Cytoskeleton organization |
| GRIP1 associated protein 1 | A2AEW8_MOUSE | Gripap1 | *-10.00* | 1.96E-03 | 7.72 | 1.00E-03 | 10.00 | 6.46E-03 | *-4.17* | 1.68E-02 | *-3.92* | 5.63E-03 | Membrane | Unknown |
| PRKCA-binding protein | PICK1_MOUSE | Pick1 | *-10.00* | 3.68E-02 | 1.70 | 2.24E-02 | 10.00 | 2.10E-02 | *-10.00* | 4.63E-02 |  |  | Cytoplasm | Nervous system |
| Tetratricopeptide repeat protein 19. mitochondrial (TPR repeat protein 19) | TTC19_MOUSE | Ttc19 | *-10.00* | 2.84E-02 | 9.70 | 1.84E-02 | 10.00 | 1.99E-02 |  |  |  |  | Mitochondrion | Metabolism |
| Coronin-2B | COR2B_MOUSE | Coro2b | *-10.00* | 1.99E-03 | 4.51 | 8.60E-04 | 10.00 | 1.44E-02 | *-7.99* | 5.50E-03 | 2.93 | 5.79E-03 | Cytoskeleton | Cytoskeleton organization |
| Isoform 2 of Calcium-activated potassium channel subunit alpha-1 | KCMA1_MOUSE | Kcnma1 | *-10.00* | 3.05E-02 | 10.00 | 2.12E-02 | 10.00 | 2.12E-02 |  |  |  |  | Membrane | Nervous System |
| Serine/threonine-protein phosphatase 2A catalytic subunit beta isoform | PP2AB_MOUSE | Ppp2cb | *-10.00* | 4.67E-02 | 6.88 | 4.92E-02 | 10.00 | 4.85E-02 |  |  |  |  | Cytoplasm | Apoptosis |
| Bcl-2-like protein 13 | B2L13_MOUSE | Bcl2l13 | *-10.00* | 2.28E-02 | 10.00 | 1.25E-02 | 10.00 | 1.27E-02 |  |  |  |  | Mitochondrion | Apoptosis |
| Nuclear autoantigenic sperm protein | NASP_MOUSE | Nasp | *-10.00* | 1.16E-03 | 10.00 | 3.81E-04 | 10.00 | 4.57E-04 | *-10.00* | 1.16E-03 | *-2.02* | 4.24E-02 | Nucleus | Cell cycle |
| Ras-related protein Rab-35 | RAB35_MOUSE | Rab35 | *-10.00* | 2.63E-02 | 10.00 | 2.45E-02 | 10.00 | 1.53E-02 | *-2.22* | 4.63E-02 |  |  | Vesicle | Endocytosis |
| Eukaryotic translation initiation factor 3 subunit K (eIF3k) | EIF3K_MOUSE | Eif3k | *-10.00* | 2.82E-02 | 5.46 | 2.02E-02 | 6.65 | 2.39E-02 | *-2.40* | 2.31E-02 |  |  | Nucleus | Translation |
| Nuclear pore complex-associated intranuclear coiled-coil protein TPR | Q7M739_MOUSE | Tpr | *-10.00* | 3.88E-02 | 10.00 | 1.95E-02 | 10.00 | 2.11E-02 |  |  |  |  | Nucleus | Transport |
| E3 ubiquitin-protein ligase RBX1 | RBX1_MOUSE | Rbx1 | *-10.00* | 2.13E-02 | 10.00 | 1.26E-02 | 10.00 | 1.22E-02 | *-2.78* | 3.98E-02 | *-9.06* | 4.05E-02 | Cytoplasm | Ubiquitination |
| Rho GTPase-activating protein 23 | RHG23_MOUSE | Arhgap23 | *-10.00* | 3.36E-02 | 10.00 | 2.48E-02 | 10.00 | 1.71E-02 |  |  |  |  | Unknown | Signaling |
| LanC-like protein 1 | LANC1_MOUSE | Lancl1 | *-10.00* | 2.15E-02 | 10.00 | 1.21E-02 | 10.00 | 1.20E-02 |  |  |  |  | Membrane | Metabolism |
| Ubiquitin carboxyl-terminal hydrolase | UBP13_MOUSE | Usp13 | *-10.00* | 2.86E-02 | 10.00 | 1.38E-02 | 10.00 | 1.38E-02 |  |  |  |  | Unknown | Ubiquitination |
| DnaJ homolog subfamily C member 7 | DNJC7_MOUSE | Dnajc7 | *-10.00* | 2.93E-02 | 10.00 | 1.41E-02 | 10.00 | 1.39E-02 |  |  |  |  | Cytoskeleton | Protein folding |
| Armadillo repeat-containing X-linked protein 2 | ARMX2_MOUSE | Armcx2 | *-10.00* | 4.86E-02 | 10.00 | 1.78E-02 | 10.00 | 1.81E-02 |  |  |  |  | Membrane | Unknown |
| Testican-2 | TICN2_MOUSE | Spock2 | *-10.00* | 3.01E-02 | 10.00 | 1.40E-02 | 10.00 | 1.41E-02 |  |  |  |  | Secreted | Ion binding |
| Sphingomyelin phosphodiesterase 3 | NSMA2_MOUSE | Smpd3 | *-10.00* | 2.32E-02 | 10.00 | 1.25E-02 | 10.00 | 1.37E-02 | *-10.00* | 4.64E-02 | *-10.00* | 3.99E-02 | Vesicle | Response |
| HEAT repeat-containing protein 5B | HTR5B_MOUSE | Heatr5b | *-10.00* | 2.75E-02 | 10.00 | 1.35E-02 | 10.00 | 1.37E-02 |  |  |  |  | Unknown | Binding |
| Laminin subunit beta-1 (Laminin B1 chain) | LAMB1_MOUSE | Lamb1 | *-10.00* | 3.54E-02 | 10.00 | 3.16E-02 | 10.00 | 3.01E-02 |  |  |  |  | Secreted | Cell adhesion |
| Vesicular integral-membrane protein VIP36 | LMAN2_MOUSE | Lman2 | *-10.00* | 2.70E-02 | 10.00 | 1.34E-02 | 10.00 | 1.34E-02 |  |  |  |  | Vesicle | Transport |
| Uncharacterized protein C1orf21 homolog | CA021_MOUSE | C1orf21 | *-10.00* | 2.56E-02 | 10.00 | 1.32E-02 | 10.00 | 1.31E-02 |  |  |  |  | Unknown | Unknown |
| Isoform 2 of PITH domain-containing protein 1 | PITH1_MOUSE | Pithd1 | *-10.00* | 2.85E-02 | 10.00 | 1.38E-02 | 10.00 | 1.39E-02 |  |  |  |  | Unknown | Unknown |
| BRI3-binding protein | BRI3B_MOUSE | Bri3bp | *-10.00* | 2.38E-02 | 10.00 | 1.26E-02 | 10.00 | 1.27E-02 |  |  |  |  | Mitochondrion | Tumorogenesis |
| Actin-related protein 2/3 complex subunit 1B | ARC1B_MOUSE | Arpc1b | *-10.00* | 2.62E-02 | 10.00 | 1.33E-02 | 3.15 | 1.72E-02 |  |  |  |  | Cytoskeleton | Cytoskeleton organization |
| Solute carrier family 37 (Glucose-6-phosphate transporter). member 4 | Q9D1F9_MOUSE | Slc37a4 | *-10.00* | 2.93E-02 | 10.00 | 1.40E-02 | 10.00 | 1.40E-02 |  |  |  |  | Cytoplasm | Transport |
| MCG125361. isoform CRA_a | D3Z742_MOUSE | 1600021P15  Rik | *-10.00* | 2.61E-02 | 10.00 | 1.32E-02 | 2.00 | 1.81E-02 |  |  | *-10.00* | 4.61E-02 | Unknown | Unknown |
| Calcium-binding and coiled-coil domain-containing protein 1 | CACO1_MOUSE | Calcoco1 | *-10.00* | 2.75E-02 | 10.00 | 1.36E-02 | 10.00 | 1.61E-02 |  |  |  |  | Nucleus | Transcription |
| Coiled-coil domain-containing protein 115 | CC115_MOUSE | Ccdc115 | *-10.00* | 3.69E-02 | 10.00 | 2.88E-02 | 10.00 | 2.50E-02 |  |  |  |  | Vesicle | Unknown |
| Syndecan-3 | SDC3_MOUSE | Sdc3 | *-10.00* | 2.42E-02 | 10.00 | 1.27E-02 | 10.00 | 1.28E-02 |  |  |  |  | Membrane | Nervous system |
| Potassium voltage-gated channel subfamily KQT member 2 | B7ZBW2_MOUSE | Kcnq2 | *-10.00* | 3.19E-02 | 10.00 | 1.93E-02 | 10.00 | 2.08E-02 |  |  |  |  | Membrane | Nervous system |
| MKIAA1243 protein (MKL/myocardin-like 2) (Fragment) | Q5DTZ3_MOUSE | Mkl2 | *-10.00* | 2.60E-02 | 10.00 | 2.18E-02 | 10.00 | 2.01E-02 | *-10.00* | 2.60E-02 |  |  | Nucleus | Transcription |
| Long-chain-fatty-acid--CoA ligase 4 | ACSL4_MOUSE | Acsl4 | *-10.00* | 3.69E-02 | 10.00 | 1.32E-02 | 2.39 | 1.80E-02 |  |  |  |  | Mitochondrion | Metabolism |
| Leucine zipper transcription factor-like protein 1 | LZTL1_MOUSE | Lztfl1 | *-10.00* | 4.05E-02 | 10.00 | 3.09E-02 | 10.00 | 3.02E-02 |  |  |  |  | Unknown | Unknown |
| Receptor-type tyrosine-protein phosphatase F (Fragment) | F6S1X8_MOUSE | Ptprf | *-10.00* | 2.77E-02 | 10.00 | 1.36E-02 | 10.00 | 1.38E-02 |  |  |  |  | Membrane | Cell adhesion |
| Integrin alpha-V | ITAV_MOUSE | Itgav | *-10.00* | 2.88E-02 | 10.00 | 1.38E-02 | 10.00 | 1.37E-02 |  |  |  |  | Membrane | Cell adhesion |
| UBX domain-containing protein 6 | UBXN6_MOUSE | Ubxn6 | *-10.00* | 2.95E-02 | 10.00 | 1.39E-02 | 10.00 | 1.38E-02 |  |  |  |  | Cytoplasm | Ubiquitination |
| Anoctamin-10 | ANO10_MOUSE | Ano10 | *-10.00* | 2.79E-02 | 10.00 | 1.36E-02 | 10.00 | 1.36E-02 |  |  |  |  | Membrane | Transport |
| Protein Etl4 | D3Z781_MOUSE | Etl4 | *-10.00* | 2.99E-02 | 10.00 | 1.41E-02 | 10.00 | 1.40E-02 |  |  |  |  | Cytoplasm | Skeletal dev |
| Protein FAM65A | FA65A_MOUSE | Fam65a | *-10.00* | 2.79E-02 | 10.00 | 1.36E-02 | 10.00 | 1.37E-02 |  |  |  |  | Cytoplasm | Unknown |
| Splicing factor 45 (45 kDa-splicing factor) (RNA-binding motif protein 17) | SPF45_MOUSE | Rbm17 | *-10.00* | 2.65E-02 | 10.00 | 1.36E-02 | 10.00 | 1.34E-02 |  |  |  |  | Nucleus | RNA processing |
| Rad23a protein (UV excision repair protein RAD23 homolog A) | Q8CAP3_MOUSE | Rad23a | *-10.00* | 2.66E-02 | 10.00 | 1.37E-02 | 10.00 | 1.58E-02 |  |  |  |  | Nucleus | Ubiquitination |
| Isoform 2 of FAD synthase | FAD1_MOUSE | Flad1 | *-10.00* | 2.59E-02 | 10.00 | 1.32E-02 | 10.00 | 1.57E-02 |  |  | *-10.00* | 4.89E-02 | Cytoplasm | Metabolism |
| U2 snRNP-associated SURP motif-containing protein (U2-associated protein SR140) | SR140_MOUSE | U2surp | *-10.00* | 2.73E-02 | 10.00 | 1.34E-02 | 10.00 | 1.57E-02 |  |  |  |  | Nucleus | RNA processing |
| Zinc finger protein 428 | E9PZE0_MOUSE | Zfp428 | *-10.00* | 2.23E-02 | 10.00 | 1.24E-02 | 10.00 | 1.47E-02 | 10.00 | 3.21E-02 | 10.00 | 4.39E-02 | Intracellular | Ion binding |
| Arfaptin-2 | ARFP2_MOUSE | Arfip2 |  |  | 9.20 | 2.65E-02 | 5.23 | 3.76E-02 |  |  |  |  | Cytoplasm | Cytoskeleton organization |
| GTP-binding protein SAR1b | SAR1B_MOUSE | Sar1b |  |  | 9.16 | 2.90E-02 | 3.15 | 3.73E-02 | 7.73 | 3.41E-02 | 2.78 | 3.53E-02 | Vesicle | Transport |
| Methylcrotonoyl-CoA carboxylase beta chain. mitochondrial | MCCB_MOUSE | Mccc2 |  |  | 8.00 | 2.89E-02 | 9.66 | 2.75E-02 | 10.00 | 3.26E-02 |  |  | Mitochondrion | Metabolism |
| Engulfment and cell motility protein 1 (Protein ced-12 homolog) | ELMO1_MOUSE | Elmo1 |  |  | 6.40 | 2.82E-02 | 8.79 | 2.71E-02 | 9.95 | 3.53E-02 | 10.00 | 3.37E-02 | cytoplasm | Cytoskeleton organization |
| Ataxin-2-like protein | Q3TGG2_MOUSE | Atxn2l |  |  | 5.86 | 2.82E-02 | 7.01 | 2.76E-02 |  |  | 10.00 | 3.12E-02 | Membrane | Unknown |
| TSC22 domain family protein 1 | D3Z0V7_MOUSE | Tsc22d1 |  |  | 5.16 | 1.30E-02 | 5.14 | 1.32E-03 |  |  |  |  | Nucleus | Apoptosis |
| Diacylglycerol kinase beta (DAG kinase beta) | F8WII9_MOUSE | Dgkb |  |  | 4.43 | 3.67E-02 | 4.45 | 3.76E-02 | 6.01 | 2.04E-03 | 7.02 | 1.76E-03 | Cytoplasm | Metabolism |
| SNW domain-containing protein 1 | SNW1_MOUSE | Snw1 |  |  | 4.39 | 9.80E-03 | 10.00 | 1.29E-03 | 2.99 | 5.17E-03 | 5.33 | 2.11E-03 | Nucleus | Transcription |
| Cysteine and glycine-rich protein 1 (CRP1) | CSRP1_MOUSE | Csrp1 |  |  | 3.90 | 2.01E-03 | 2.10 | 2.83E-03 |  |  |  |  | Nucleus | Cytoskeleton organization |
| CDC23 (Cell division cycle 23. yeast. homolog). isoform CRA_a | G3X8W7_MOUSE | Cdc23 |  |  | 3.66 | 2.96E-02 | 9.52 | 2.73E-02 |  |  |  |  | Nucleus | Cell division |
| Misshapen-like kinase 1 (Fragment) | F7AMS7_MOUSE | Mink1 |  |  | 3.11 | 2.52E-02 | 4.02 | 2.87E-02 |  |  |  |  | Cytoplasm | Nervous system |
| MCG14657 | G3XA56_MOUSE | mCG_14657 |  |  | 3.03 | 2.81E-02 | 4.27 | 2.79E-02 | 10.00 | 2.93E-02 |  |  | xxxxx | Unknown |
| Isoform HMG-Y of High mobility group protein HMG-I/HMG-Y | HMGA1_MOUSE | Hmga1 |  |  | 3.00 | 1.97E-02 | 4.02 | 1.68E-02 |  |  |  |  | Nucleus | Transcription |
| MAP7 domain-containing protein 1 | MA7D1_MOUSE | Map7d1 |  |  | 2.54 | 3.53E-02 | 10.00 | 2.56E-02 |  |  | 7.48 | 3.47E-02 | Cytoskeleton | Unknown |
| Echinoderm microtubule-associated protein-like 2 | E9QK48_MOUSE | Eml2 |  |  | 2.35 | 2.81E-02 | 1.78 | 2.76E-02 |  |  | *-4.85* | 3.00E-02 | Cytoplasm | Metabolism |
| ProSAAS (IA-4) (Proprotein convertase subtilisin/kexin type 1 inhibitor) | PCSK1_MOUSE | Pcsk1n |  |  | 2.30 | 1.60E-02 | 1.85 | 7.40E-03 |  |  | *-1.93* | 5.51E-03 | Secreted | Nervous System |
| Lipid phosphate phosphohydrolase 3 (Phosphatidic acid phosphatase 2b) | LPP3_MOUSE | Ppap2b |  |  | 2.18 | 3.34E-02 | 1.30 | 2.63E-02 | 1.70 | 4.13E-02 |  |  | Vesicle | Metabolism |
| ADP-ribosylation factor-like protein 15 (ARF-related protein 2) | ARL15_MOUSE | Arl15 |  |  | 1.90 | 2.40E-02 | 1.44 | 2.44E-02 | 4.25 | 3.78E-02 | 6.32 | 2.98E-02 | Unknown | Signaling |
| Cysteine desulfurase. mitochondrial (Nitrogen fixation gene 1 (S. cerevisiae)) | Q8C6I5_MOUSE | Nfs1 |  |  | 1.85 | 4.40E-02 | 8.75 | 2.70E-02 |  |  | 8.88 | 3.06E-02 | Cytoplasm | Metabolism |
| Lamin-B2 | LMNB2_MOUSE | Lmnb2 |  |  | 1.60 | 3.10E-02 | 1.62 | 2.75E-02 | 1.55 | 3.26E-02 |  |  | Nucleus | Apoptosis |
| NADH dehydrogenase [ubiquinone] 1 beta subcomplex subunit 11. mitochondrial (Complex I-ESSS) (Neuronal protein 15.6) | NDUBB_MOUSE | Ndufb11 |  |  | 1.49 | 1.30E-03 | 1.51 | 1.50E-03 | *-1.74* | 2.77E-03 | *-2.16* | 1.51E-02 | Mitochondrion | Transport |
| F-actin-capping protein subunit alpha-1 | CAZA1_MOUSE | Capza1 |  |  | *-1.32* | 3.67E-02 | 1.47 | 2.61E-02 |  |  |  |  | Cytoskeleton | Cytoskeleton organization |
| Low-density lipoprotein receptor-related protein 1B | E9Q202_MOUSE | Lrp1b |  |  | *-5.86* | 3.56E-02 | 2.37 | 2.43E-02 | *-6.01* | 3.56E-02 | 2.08 | 2.47E-02 | Membrane | Receptor |
| Isoform 2 of Protein phosphatase 1 regulatory subunit 12A | MYPT1_MOUSE | Ppp1r12a | 2.76 | 8.90E-03 |  |  | 1.63 | 1.26E-02 | 3.67 | 1.34E-02 | 3.63 | 1.35E-02 | Cytoplasm | Cytoskeleton organization |
| G protein pathway suppressor 1 | Q3MIA8_MOUSE | Gps1 | 1.77 | 1.96E-02 |  |  | *-2.01* | 6.55E-03 | 1.81 | 1.54E-02 | 1.64 | 1.85E-02 | Nucleus | Ubiquitination |
| MKIAA1065 protein (Fragment) | Q69ZS5_MOUSE | Epn2 | 1.72 | 3.75E-03 |  |  | *-1.33* | 1.07E-02 | 1.75 | 4.29E-03 | 1.50 | 4.77E-03 | Cytoplasm | Endocytosis |
| DBIRD complex subunit ZNF326 (Zinc finger protein 326) (Zinc finger protein-associated with nuclear matrix of 75 kDa) | ZN326_MOUSE | Znf326 | *-1.37* | 2.18E-03 |  |  | 1.57 | 4.75E-04 | *-1.34* | 1.30E-02 | 2.54 | 2.38E-03 | Nucleus | Transcription |
| Mitochondrial import receptor subunit TOM20 homolog | TOM20_MOUSE | Tomm20 | *-2.67* | 2.21E-02 |  |  | 3.46 | 1.93E-03 | *-3.09* | 1.51E-02 | 2.44 | 3.61E-03 | Mitochondrion | Transport |
| Isoform 2B of GTPase KRas | RASK_MOUSE | Kras | -7.45 | 5.17E-03 |  |  | 1.72 | 2.48E-03 | *-7.54* | 3.72E-03 | *-5.24* | 2.13E-02 | Membrane | Nervous system |
| Map4k4 protein | B7ZNR9_MOUSE | Map4k4 |  |  |  |  | 9.69 | 2.65E-02 |  |  | 10.00 | 3.11E-02 | Cytoplasm | Response |
| Influenza virus NS1A-binding protein homolog (NS1-BP) (NS1-binding protein homolog) | NS1BP_MOUSE | Ivns1abp |  |  |  |  | 1.92 | 2.85E-02 |  |  |  |  | Nucleus | Response |
| Mitotic checkpoint protein BUB3 (WD repeat type I transmembrane protein A72.5) | BUB3_MOUSE | Bub3 |  |  |  |  | 1.67 | 1.92E-02 |  |  | 1.93 | 3.80E-02 | Nucleus | cell division |
| Vasohibin-1 | VASH1_MOUSE | Vash1 |  |  |  |  | 1.56 | 2.77E-02 |  |  | 4.21 | 2.98E-02 | Secreted | Cell cycle |
| Serine/arginine-rich splicing factor 10 (Neural-salient serine/arginine-rich protein) (Neural-specific SR protein) | SRS10_MOUSE | Srsf10 |  |  |  |  | 1.32 | 1.15E-03 |  |  | 1.31 | 1.84E-03 | Nucleus | Transcription |
| Serine/threonine-protein phosphatase 6 catalytic subunit (PP6C) (EC 3.1.3.16) | PPP6_MOUSE | Ppp6c |  |  |  |  | 1.31 | 2.02E-03 |  |  |  |  | Cytoplasm | Cell cycle |
| Cell cycle control protein 50A | CC50A_MOUSE | Tmem30a | 3.30 | 1.65E-03 | *-3.05* | 1.10E-02 |  |  | 1.93 | 1.25E-02 | 3.31 | 1.72E-03 | Membrane | xxxxxx |
| GDP-L-fucose synthase | FCL_MOUSE | Tsta3 | 1.45 | 3.69E-03 | *-1.34* | 1.68E-02 |  |  |  |  | 1.64 | 2.99E-03 | Membrane | Metabolism |
| Thioredoxin-related transmembrane protein 1 | TMX1_MOUSE | Tmx1 | *-1.42* | 1.86E-02 | 1.88 | 8.46E-04 |  |  |  |  | *-1.43* | 1.04E-02 | Membrane | Apoptosis |
| Annexin A2 | ANXA2_MOUSE | Anxa2 | *-3.22* | 7.17E-03 | 3.60 | 1.63E-03 |  |  | 1.70 | 1.22E-02 | *-2.45* | 6.73E-03 | Membrane | Cytoskeleton organization |
| Apolipoprotein C-I | APOC1_MOUSE | Apoc1 |  |  | 2.47 | 3.70E-02 |  |  |  |  |  |  | Secreted | Transport |
| NADH dehydrogenase [ubiquinone] 1 beta subcomplex subunit 8. mitochondrial | NDUB8_MOUSE | Ndufb8 |  |  | 1.47 | 1.76E-03 |  |  | 1.36 | 1.41E-02 | -1.50 | 1.74E-02 | Mitochondrion | Metabolism |
| Angiotensin-converting enzyme | ACE_MOUSE | Ace | 10.00 | 2.64E-02 |  |  |  |  | 10.00 | 2.34E-02 | 10.00 | 2.52E-02 | Membrane | Nervous system |
| E3 ubiquitin-protein ligase RNF213 (EC 6.3.2.-) | RN213_MOUSE | Rnf213 | 10.00 | 1.32E-02 |  |  |  |  | 10.00 | 9.98E-03 | 10.00 | 1.32E-02 | Cytoplasm | Ubiquitination |
| Immunity-related GTPase family M protein 1 | IRGM1_MOUSE | Irgm1 | 6.51 | 1.50E-03 |  |  |  |  | 6.76 | 1.51E-03 | 7.12 | 1.49E-03 | Vesicle | Response |
| Isoform 3 of Serine/arginine repetitive matrix protein 2 | SRRM2_MOUSE | Srrm2 | 5.87 | 2.07E-03 |  |  |  |  | 7.19 | 9.51E-03 | 6.46 | 1.61E-02 | Nucleus | RNA processing |
| Isoform 4 of AT-rich interactive domain-containing protein 1A | ARI1A_MOUSE | Arid1a | 2.28 | 9.61E-03 |  |  |  |  | 1.55 | 1.28E-02 | 2.94 | 1.52E-02 | Nucleus | Transcription |
| UBX domain-containing protein 4 | UBXN4_MOUSE | Ubxn4 | 1.61 | 3.37E-02 |  |  |  |  | 1.49 | 3.40E-02 | 1.57 | 3.07E-02 | Membrane | Response |
| Adenine phosphoribosyl transferase | Q6PK77_MOUSE | Aprt | 1.45 | 2.61E-03 |  |  |  |  |  |  | 1.43 | 2.19E-03 | Cytoplasm | Metabolism |
| Protein Tmed7 | D3YZZ5_MOUSE | Tmed7 | *-1.33* | 4.28E-02 |  |  |  |  | *-1.31* | 3.43E-02 |  |  | Membrane | Transport |
| Brain protein 44-like protein | MPC1_MOUSE | Brp44l | *-1.47* | 4.10E-02 |  |  |  |  |  |  | *-1.87* | 3.78E-02 | Mitochondrion | Transport |
| ATP-dependent RNA helicase DDX39A | DX39A_MOUSE | Ddx39a | *-1.59* | 3.37E-03 |  |  |  |  | 1.31 | 1.21E-02 | 2.02 | 3.52E-03 | Nucleus | RNA processing |
| Protein C19orf12 homolog | CS012_MOUSE |  |  |  |  |  |  |  | 10.00 | 2.05E-02 | 10.00 | 2.00E-02 | Mitochondrion | Unknown |
| Protein Ildr2 | E9Q9U5_MOUSE | Ildr2 |  |  |  |  |  |  | 10.00 | 3.00E-02 | *-6.37* | 2.65E-02 | Unknown | Development |
| Vacuolar fusion protein CCZ1 homolog | CCZ1_MOUSE | Ccz1 |  |  |  |  |  |  | 7.25 | 3.32E-02 | 10.00 | 2.74E-02 | Vesicle | Unknown |
| OCIA domain-containing protein 1 | OCAD1_MOUSE | Ociad1 |  |  |  |  |  |  | 5.38 | 2.65E-02 | 8.19 | 2.89E-02 | Vesicle | Unknown |
| Translocation protein SEC63 homolog | SEC63_MOUSE | Sec63 |  |  |  |  |  |  | 5.11 | 3.27E-02 | 5.09 | 3.41E-02 | Vesicle | Protein folding |
| STE20/SPS1-related proline-alanine-rich protein kinase | STK39_MOUSE | Stk39 |  |  |  |  |  |  | 3.98 | 1.21E-02 | 2.40 | 2.61E-03 | Cytoplasm | Cytoskeleton organization |
| Isoform 3 of Dynamin-1 | DYN1_MOUSE | Dnm1 |  |  |  |  |  |  | 3.46 | 2.48E-02 | 8.30 | 2.41E-02 | Cytoskeleton | Cytoskeleton organization |
| Isoform 2 of Seizure protein 6 | SEZ6_MOUSE | Sez6 |  |  |  |  |  |  | 3.37 | 8.00E-04 | 2.83 | 1.71E-02 | Membrane | Nervous systemem |
| Septin-6 | SEPT6_MOUSE | sept6 |  |  |  |  |  |  | 3.02 | 2.31E-02 | 2.22 | 2.71E-02 | Cytoskeleton | Nervous systemem |
| Guanylate-binding protein 4 | GBP4_MOUSE | Gbp4 |  |  |  |  |  |  | 2.66 | 1.66E-02 | 4.13 | 1.37E-02 | Cytoplasm | Response |
| Serine/arginine-rich splicing factor 5 | SRSF5_MOUSE | Srsf5 |  |  |  |  |  |  | 1.50 | 4.04E-03 |  |  | Nucleus | Transcription |
| Metaxin-2 | MTX2_MOUSE | Mtx2 |  |  |  |  |  |  | 1.32 | 1.43E-02 | 1.38 | 4.27E-03 | Mitochondrion | Unknown |
| U1 small nuclear ribonucleoprotein 70 kDa | RU17_MOUSE | Snrnp70 |  |  |  |  |  |  | *-1.76* | 1.04E-02 | 1.79 | 1.85E-02 | Nucleus | RNA processing |
| MKIAA0655 protein (Fragment) | Q6ZQ77_MOUSE | Hip1r |  |  |  |  |  |  | *-1.89* | 3.30E-03 | *-2.23* | 1.78E-02 | Unknown | Endocytosis |
| Regulatory-associated protein of mTOR | RPTOR_MOUSE | Rptor |  |  |  |  |  |  | *-3.38* | 1.88E-02 |  |  | Cytoplasm | Response |
| Fibrinogen gamma chain | Q3UER8_MOUSE | Fgg |  |  |  |  |  |  | *-10.00* | 2.60E-02 | *-10.00* | 2.43E-02 | Unknown | Signaling |
| DnaJ (Hsp40) homolog. subfamily C. member 19. isoform CRA_c | G3X8S1_MOUSE | Dnajc19 |  |  |  |  |  |  | *-10.00* | 2.91E-02 |  |  | Unknown | Development |
