# Supplementary material for: Kinetic Analysis of Mouse Brain Proteome Alterations Following Chikungunya Virus Infection before and after Appearance of Clinical Symptoms
Source: PLoS One. 2014 Mar 11;9(3):e91397. doi: 10.1371/journal.pone.0091397 (PMC3949995; doi:10.1371/journal.pone.0091397)
Supplement: Table S6 — Ingenuity Canonical Pathways showing a strong significant association [-Log(p-value) >2.0] using the total dataset of 177 proteins differentially expressed in the 3 observations (early (E) vs mock (M), late paralytic (LP) vs E and late tetanus-like (LT) vs E). (DOC) [file pone.0091397.s007.doc]

**Table S6.** Ingenuity Canonical Pathways showing a strong significant association [-Log(p-value) >2.0] using the total dataset of 177 proteins differentially expressed in the 3 observations (early (E) vs mock (M), late paralytic (LP) vs E and late tetanus-like (LT) vs E).

| **Ingenuity Canonical Pathways** | **-log(p-value)** | **Molecules** |
| --- | --- | --- |
| Sertoli Cell-Sertoli Cell Junction Signaling | 5.19 | ITGB1, SPTBN1, EPN2, TUBB3, NRAS, SORBS1, PPAP2B, KRAS, SPTAN1, PTEN |
| CDK5 Signaling | 3.77 | ITGB1, PPP2CB, NRAS, LAMB1, PPP1R12A, KRAS |
| Actin Nucleation by ARP-WASP Complex | 3.75 | ITGB1, NRAS, ARPC1B, PPP1R12A, KRAS |
| Epithelial Adherens Junction Signaling | 3.36 | EPN2, TUBB3, NRAS, ARPC1B, SORBS1, KRAS, PTEN |
| Integrin Signaling | 3.27 | ITGB1, NRAS, ARPC1B, ARF4, ITGAV, PPP1R12A, KRAS, PTEN |
| Role of Tissue Factor in Cancer | 3.22 | ITGB1, NRAS, ARRB1, ITGAV, KRAS, PTEN |
| Germ Cell-Sertoli Cell Junction Signaling | 3.18 | ITGB1, EPN2, TUBB3, NRAS, SORBS1, PPAP2B, KRAS |
| Semaphorin Signaling in Neurons | 2.84 | ITGB1, DPYSL2, CRMP1, DPYSL3 |
| Neuregulin Signaling | 2.84 | ITGB1, PICK1, NRAS, KRAS, PTEN |
| Insulin Receptor Signaling | 2.81 | NRAS, PPP1R12A, KRAS, ACLY, PTPRF, PTEN |
| Clathrin-mediated Endocytosis Signaling | 2.75 | ITGB1, APOC1, DNM1, ALB, ARRB1, ARPC1B, APOA2 |
| Paxillin Signaling | 2.60 | ITGB1, NRAS, ARFIP2, ITGAV, KRAS |
| Regulation of eIF4 and p70S6K Signaling | 2.56 | ITGB1, PPP2CB, NRAS, EIF3H, KRAS, EIF3K |
| Rac Signaling | 2.54 | ITGB1, NRAS, ARFIP2, ARPC1B, KRAS |
| Agrin Interactions at Neuromuscular Junction | 2.42 | ITGB1, NRAS, LAMB1, KRAS |
| NF-κB Activation by Viruses | 2.31 | ITGB1, NRAS, ITGAV, KRAS |
| PI3K/AKT Signaling | 2.21 | ITGB1, PPP2CB, NRAS, KRAS, PTEN |
| Ceramide Signaling | 2.17 | PPP2CB, NRAS, KRAS, SMPD3 |
| Melanoma Signaling | 2.15 | NRAS, KRAS, PTEN |
| UVC-Induced MAPK Signaling | 2.15 | NRAS, KRAS, SMPD3 |
| Colanic Acid Building Blocks Biosynthesis | 2.13 | TSTA3, UGP2 |
| FAK Signaling | 2.04 | ITGB1, NRAS, KRAS, PTEN |
| Acetyl-CoA Biosynthesis III (from Citrate) | 2.03 | ACLY |
| Alanine Biosynthesis III | 2.03 | NFS1 |
| GABA Receptor Signaling | 2.01 | DNM1, NSF, GABRA1 |
| Virus Entry via Endocytic Pathways | 2.01 | ITGB1, DNM1, NRAS, KRAS |
